# The single berberine bridge enzyme homolog of *Physcomitrella patens* is a cellobiose oxidase

**DOI:** 10.1111/febs.14458

**Published:** 2018-04-19

**Authors:** Marina Toplak, Gertrud Wiedemann, Jelena Ulićević, Bastian Daniel, Sebastian N. W. Hoernstein, Jennifer Kothe, Johannes Niederhauser, Ralf Reski, Andreas Winkler, Peter Macheroux

**Affiliations:** ^1^ Institute of Biochemistry Graz University of Technology Austria; ^2^ Plant Biotechnology Faculty of Biology University of Freiburg Germany; ^3^ BIOSS Centre for Biological Signalling Studies University of Freiburg Germany

**Keywords:** enzyme catalysis, enzyme mechanism, flavin adenine dinucleotide, plant biochemistry, protein structure

## Abstract

The berberine bridge enzyme from the California poppy *Eschscholzia californica* (*Ec* BBE) catalyzes the oxidative cyclization of (*S*)‐reticuline to (*S*)‐scoulerine, that is, the formation of the berberine bridge in the biosynthesis of benzylisoquinoline alkaloids. Interestingly, a large number of *BBE‐*like genes have been identified in plants that lack alkaloid biosynthesis. This finding raised the question of the primordial role of *BBE* in the plant kingdom, which prompted us to investigate the closest relative of *Ec* BBE in *Physcomitrella patens* (*Pp* BBE1), the most basal plant harboring a *BBE‐*like gene. Here, we report the biochemical, structural, and *in vivo* characterization of *Pp* BBE1. Our studies revealed that *Pp* BBE1 is structurally and biochemically very similar to *Ec* BBE. In contrast to *Ec* BBE, we found that *Pp* BBE1 catalyzes the oxidation of the disaccharide cellobiose to the corresponding lactone, that is, *Pp* BBE1 is a cellobiose oxidase. The enzymatic reaction mechanism was characterized by a structure‐guided mutagenesis approach that enabled us to assign a catalytic role to amino acid residues in the active site of *Pp* BBE1. *In vivo* experiments revealed the highest level of *PpBBE1* expression in chloronema, the earliest stage of the plant's life cycle, where carbon metabolism is strongly upregulated. It was also shown that the enzyme is secreted to the extracellular space, where it may be involved in later steps of cellulose degradation, thereby allowing the moss to make use of cellulose for energy production. Overall, our results suggest that the primordial role of BBE‐like enzymes in plants revolved around primary metabolic reactions in carbohydrate utilization.

**Database:**

Structural data are available in the PDB under the accession numbers 6EO4 and 6EO5.

**Abbreviations**
BBE: berberine bridge enzyme
GOOX: glucooligosaccharide oxidase
*Pp*BBE1: berberine bridge enzyme homolog from *Physcomitrella patens* (gene #1)
THCA: Δ^1^‐tetrahydrocannabinolic acid

## Introduction

The berberine bridge enzyme (BBE)‐like protein family is a large enzyme family found in bacteria, fungi, and plants, named after its best characterized member, the BBE from *Eschscholzia californica* (*Ec*BBE, http://www.chem.qmul.ac.uk/iubmb/enzyme/EC1/21/3/3.html). In Californian poppy (*E. californica*), BBE participates in the biosynthesis of benzylisoquinoline alkaloids, where it catalyzes the oxidative formation of the berberine bridge resulting in the conversion of (*S*)‐reticuline to (*S*)‐scoulerine 1. Whole‐genome sequencing efforts have revealed the occurrence of many *BBE*‐like genes in virtually all plants even those that lack alkaloid biosynthesis 2. In the model plant *Arabidopsis thaliana*, for example, 27 genes were identified to encode BBE‐like enzymes, with an even higher number found in *Citrus clementina* (41 genes), *Glycine max* (43 genes), and *Populus trichocarpa* (65 genes). Despite the ubiquitous and abundant presence of BBE‐like proteins in the plant kingdom, the biochemical and physiological function of the enzymes remain largely unknown. Based on multiple sequence alignments, the 27 BBE homologs of *A. thaliana* were classified into seven subfamilies 3. Recent studies have successfully assigned a function to two of these subfamilies, demonstrating oxidation of indole cyanohydrins and monolignols to the corresponding keto and aldehyde products, respectively 3, 4. Besides, earlier reports on enzymes involved in plant defense have implicated BBE‐like proteins in the oxidation of mono‐ and polysaccharides derived from glucose as well as from galactose 5. A similar activity was also assigned to the pollen allergen Phl p 4 6 and to nectarin 5, a BBE‐like protein from ornamental tobacco 7.

Unlike most other plants, the genome of the moss *Physcomitrella patens* harbors only two genes that can be assigned to the BBE‐like family 8. Gene #1 (*Pp*3c12_2640V3.1) shares the highest sequence identity with fungal carbohydrate oxidases (ca. 30% identity on the amino acid sequence level) and is closely related to *Ec*BBE and other plant enzymes from the BBE‐like family (25–28% identity on the amino acid sequence level). In contrast, gene #2 (*Pp*3c2_12420V3.1) has a much lower similarity to *Ec*BBE (< 20% sequence identity) and is more closely related to the ecdysteroid‐22‐oxidase from *Metarhizium rileyi* (29.3%; Uniprot: http://www.uniprot.org/uniprot/I0J0L0).

Because of the role of *P. patens* as a paradigm for the conquest of land by plants 9, we reasoned that unraveling the function of the moss BBE‐like protein would provide insight into the evolutionary origin of this ubiquitous multigene family in plants. Toward this goal, we have produced the BBE homolog, termed *Pp*BBE1 and encoded by gene #1 (*Pp*3c12_2640V3.1), in the methylotrophic yeast *Komagataella phaffii* and subsequently characterized the purified enzyme biochemically and structurally. In addition, expression and secretion of the wild‐type protein as well as the phenotypical characteristics of a knockout strain were studied in order to reveal the *in vivo* function of the enzyme. Here, we report that *Pp*BBE1 is an efficient cellobiose oxidase that is very similar to fungal BBE‐like oxidases, such as glucooligosaccharide oxidase (GOOX), in terms of the active site composition as well as the enzymatic reaction mechanism. Our *in vitro* findings are further supported by the data obtained from *in vivo* experiments since the highest level of expression could be detected in chloronema, an early stage of the plant's life cycle, where carbohydrate metabolism is strongly upregulated 10, 11. Thus, our results suggest that the diversity of enzymatic reactions found in the BBE‐like protein family in the plant kingdom originated from this ‘primordial’ carbohydrate oxidase.

## Results

### Gene organization, expression, and secretion of *Physcomitrella patens* BBEs

**Figure 1:**
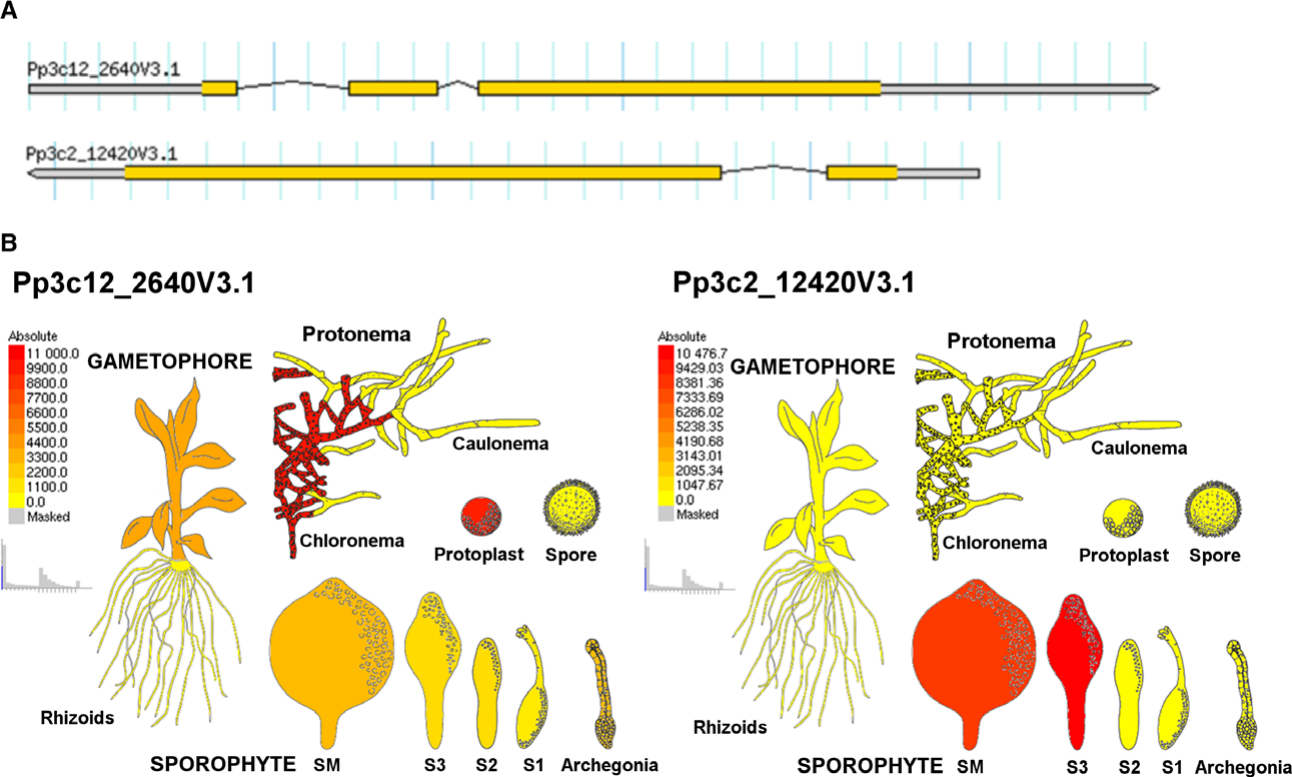
Genomic organization and expression of *PpBBE1* and *PpBBE3* throughout the life cycle. (A) *PpBBE1* is represented by the model Pp3c12_2640V3.1 and *PpBBE2* by the model Pp3c2_12420V3.1 in the latest release of the genome V3.3 available on cosmoss.org. (B) *PpBBE1* (left) and *PpBBE3* (right) expression throughout the whole lifecycle of *Physcomitrella patens* visualized using the *Physcomitrella* eFP Browser available on http://bar.utoronto.ca/efp_physcomitrella/cgi-bin/efpWeb.cgi 12.

According to the latest release of the genome V3.3 available on cosmoss.org, *PpBBE1* is located on chromosome 12 and represented by the gene model *Pp*3c12_2640V3.1 encoding a protein of 501 amino acids. The full‐length gene comprises 3243 bp, of which 2808 bp belong to the coding sequence. The latter consists of a 5′‐UTR of 498 bp, a transcript of 1506 bp and a 3′‐UTR of 804 bp, with the transcript being formed by three exons (99, 256, and 1151 bp for exon 1, 2, and 3, respectively) and two introns. *PpBBE3* (note that *PpBBE2* was identified as a pseudogene) is located on chromosome 2 and represented by *Pp*3c2_12420V3.1, consisting of two exons and an intron, encoding a protein of 586 amino acids (Fig. 1A).

The expression of *PpBBE1* throughout the whole life cycle and upon different growth conditions was analyzed *in silico* using publicly available transcriptomic data based on microarray experiments 12, 13. *PpBBE1* is highly expressed in protoplasts and chloronema cells, which both harbor large and numerous chloroplasts. Analysis of the caulonema, the fast‐growing cell type of protonema from which buds arise and which is characterized by fewer chloroplasts and slimmer cells with oblique cell walls, in contrast, did not reveal any expression. In the gametophore, expression of *PpBBE1* was restricted to the green parts, whereas in rhizoids, no expression was detected. During sporophyte development, *PpBBE1* expression was only detectable in unfertilized archegonia, the female sexual organs, and the mature spore capsule while *PpBBE3* expression was restricted to the late stages of sporophyte development (12; Fig. 1B). On dedifferentiation of leaves to chloronema as well as after incubation in darkness, *Pp*BBE1 expression was upregulated 13.

*Pp*BBE1 is about 54 kDa and harbors a predicted N‐terminal signal peptide (1–30) according to SignalP 14 as well as an internal FAD‐binding domain (78–216, PF01565) and a C‐terminal BBE domain (457–499, PF08031) according to the PFAM protein domain annotation 15. Furthermore, the *Pp*BBE1 was identified in the supernatant of *P. patens* liquid cultures by mass spectrometry whereas *Pp*BBE3 was not detected.

### Production and purification of PpBBE1 and basic characterization

**Figure 2:**
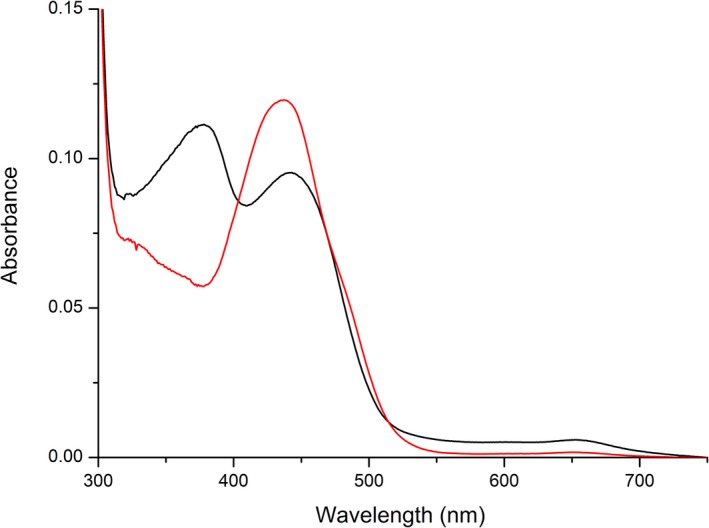
UV‐Vis absorption spectrum of native (black line) and denatured (red line) *Pp* BBE1. *Pp* BBE1 was diluted to a final concentration of about 10 μm with 50 mm HEPES buffer pH 6, before recording the spectra at 25 °C.

**Figure 3:**
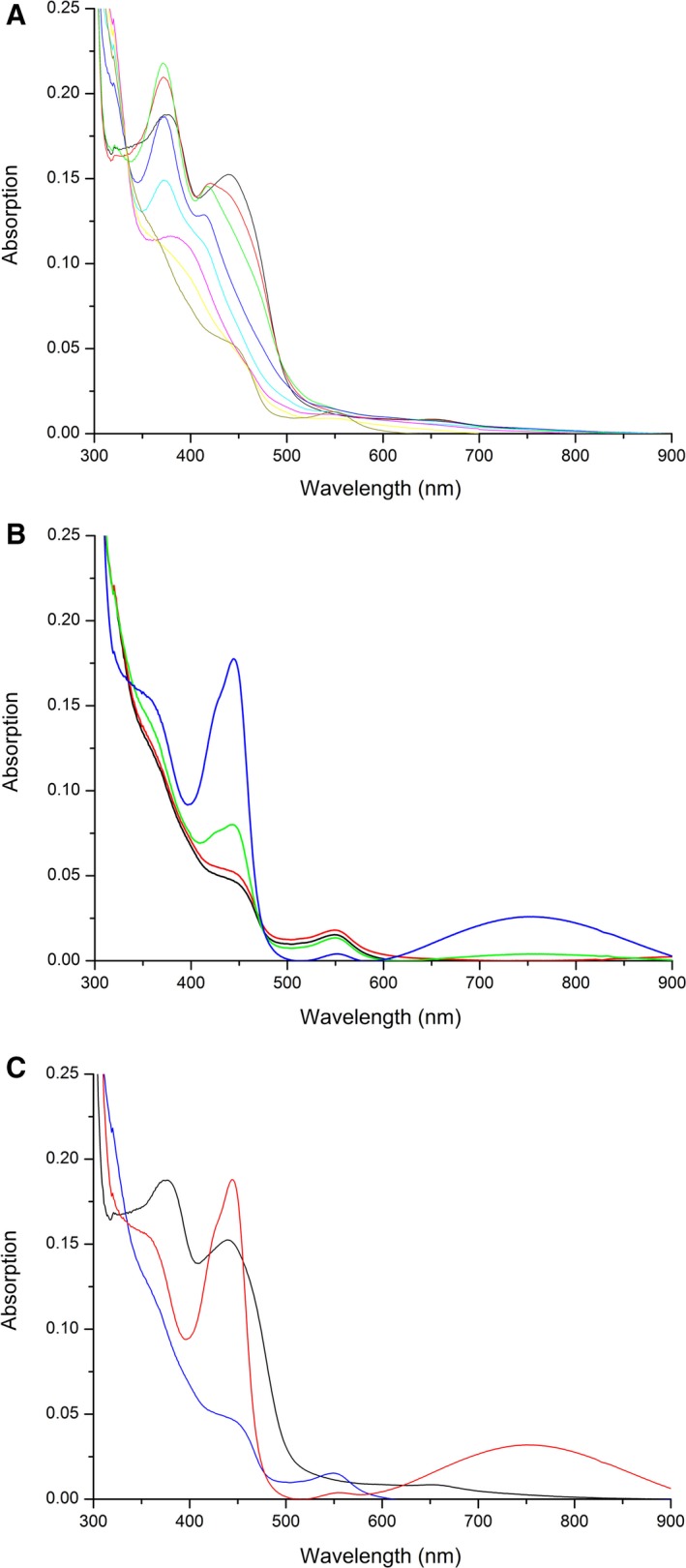
Photoreduction in *Pp* BBE1 under anoxic conditions. (A) UV‐Vis absorption spectra of *Pp* BBE1, diluted to a final concentration of about 15 μm with 50 mm HEPES pH 7, were recorded during anaerobic photoreduction. The strong increase in absorption at 371 nm within the first minutes indicates the formation of an anionic flavin semiquinone [compare start spectrum (black) with spectrum recorded after 4 min (green line)], which was subsequently reduced to the flavin hydroquinone (brown line); (B) Reoxidation of *Pp* BBE1 following photoreduction. The broad peak observed in the spectrum after full reoxidation (blue line) between 600 and 900 nm indicates the formation of 6‐thio‐FAD; (C) Comparison of the UV‐Vis absorption spectra of native (black), fully reduced (blue), and fully reoxidized (red) *Pp*BBE1.

**Figure 4:**
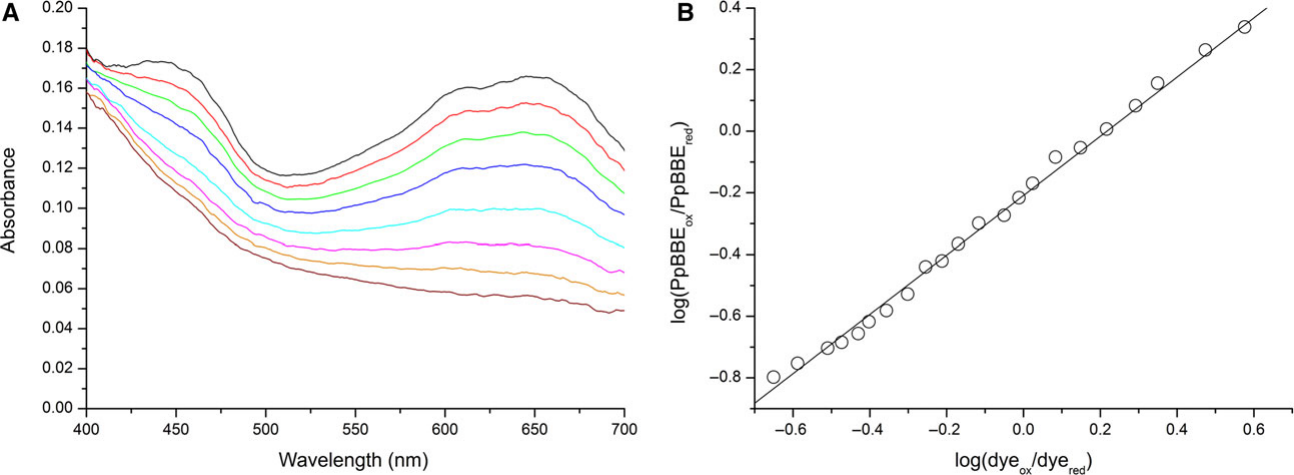
Determination of the redox potential of *Pp* BBE1 (in 50 mm HEPES pH 7). (A) The UV‐Vis absorption spectra recorded during the reduction reaction. (B) Nernst plot obtained from the experimental data, that is, log(*Pp* BBE1_ox_/*Pp* BBE1_red_) vs the log(dye_ox_/dye_red_). The slope of near unity (0.96) is in keeping with a two‐electron transfer process for both enzyme and the dye; the intercept was used to calculate the redox potential of *Pp* BBE1 (+121 ± 1 mV from three determinations).

The gene of the *P. patens* BBE‐like protein encoded by locus *Pp*3c12_2640V3.1 (gene #1) was expressed in the methylotrophic yeast *Komagataella paffii* as described in Experimental procedures. The protein (molecular mass 54 kDa) was secreted into the culture medium and purified by Ni‐NTA affinity chromatography using the C‐terminal octahistidine tag. Typical yields of pure protein varied between 5 and 7 mg·L^−1^ of culture. The UV‐Vis absorption spectrum of purified *Pp*BBE1 exhibited two peaks at 375 and 443 nm indicating the presence of a flavin chromophore (Fig. 2, black line). Denaturation of the protein resulted in a single peak at 440 nm (Fig. 2, red line). Similar spectral changes were reported for other members of the BBE family and reflect the bicovalent linkage of the isoalloxazine ring via the C6‐ and 8α‐position to a cysteine and histidine residue, respectively 2, 16, 17, 18. As both residues are conserved in the sequence of *Pp*BBE1 (H111 and C172), the observed spectral properties are in accordance with the formation of both covalent linkages. To further substantiate the presence of two covalent linkages, the protein was photoreduced under anoxic conditions. As shown in Fig. 3, reduction initially yielded the anionic flavin semiquinone, which was then further reduced to the flavin hydroquinone (Fig. 3A,B). Reoxidation of the fully reduced sample yielded absorption characteristics typical for a 6‐thioflavin with a sharp peak at 425 nm and a broad absorption centered at 750 nm (Fig. 3B,C). The underlying cleavage of the carbon–sulfur bond in the covalently linked cysteine residue was previously observed for *Ec*BBE 16 and appears to occur in all bicovalently linked flavins found in the BBE family. Yet, another hallmark of bicovalently linked flavins is their unusually high redox potential, which is typically more positive than +100 mV, for example, +132 ± 4 mV for *Ec*BBE 17. Thus, we also determined the redox potential for *Pp*BBE1 using the dye equilibration method 19. As shown in Fig. 4, the reporting dye and the flavin were reduced synchronously allowing to plot the log of the ratio of oxidized vs reduced dye against the log of the ratio of oxidized vs reduced enzyme‐linked FAD (Fig. 4A,B). From this Nernst plot, a redox potential of +121 ± 1 mV was obtained, which is comparable with other BBE‐like enzymes 2, 17, 20.

### Identification of substrates

**Figure 5:**
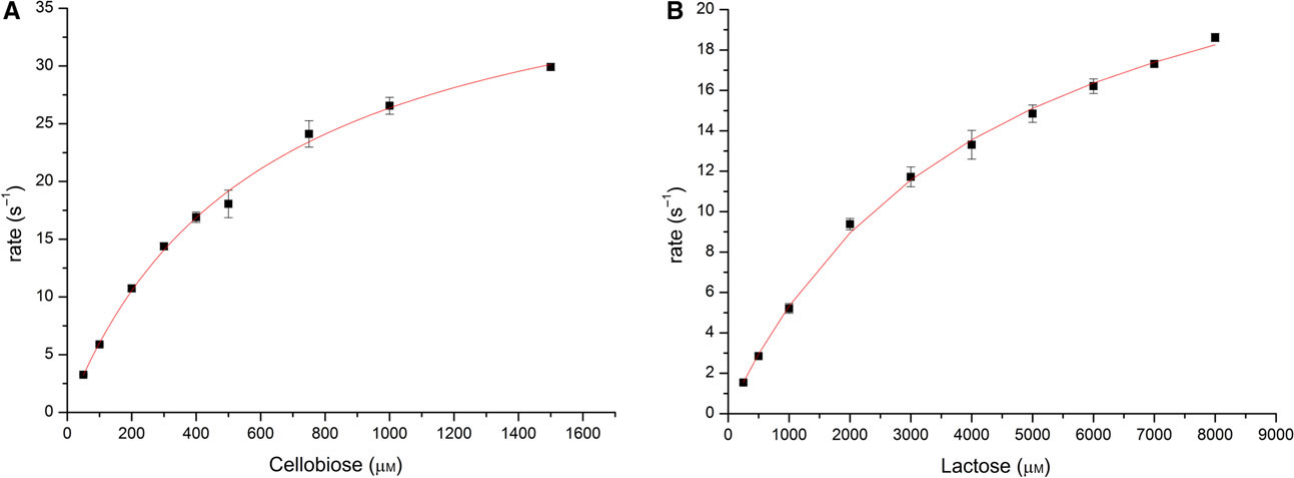
Steady‐state kinetics performed with *Pp* BBE1 using cellobiose (A) and lactose (B) as substrates. Reaction rates were determined at pH 6, near the pH‐optimum of *Pp* BBE1 wild‐type, keeping the enzyme concentration constant (10 nm) and varying the substrate concentration (cellobiose: 50–1500 μm; lactose: 250–8000 μm). From these data, it can be concluded that *Pp* BBE1 shows an about 10‐fold higher catalytic efficiency with cellobiose than with lactose (apparent *k*_cat_/*K*_M_ = 8 × 10^4^ ± 7 × 10^3^ and 7 × 10^3^ ± 1 × 10^3^ m ^−1^·s^−1^ from three determinations, respectively; the standard deviations are displayed as error bars).

**Figure 6:**
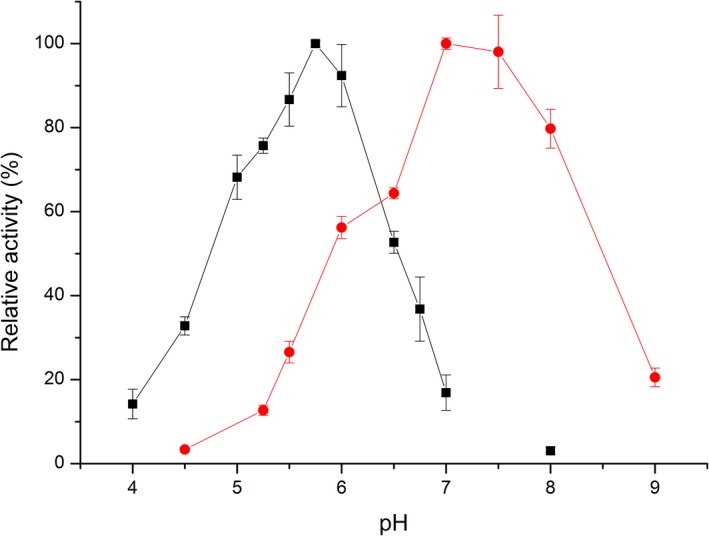
Comparison of the pH‐profile of wild‐type *Pp* BBE1 (black) and the D396A variant (red). Reaction rates were determined in the pH‐range from 4 to 8, using cellobiose (500 μm) as substrate and DCPIP (100 μm) as final electron acceptor. All reactions were started upon addition of the enzyme (wild‐type: 10 nm; D396A: 200 nm) and the decrease in absorption at 600 nm was monitored for 120 s. The wild‐type enzyme showed highest activity at pH 5.8, whereas the pH‐optimum of the variant was shifted by more than one pH‐unit to pH ~ 7.2 (turnover rates at the different pH‐values were determined in triplicates; the standard deviations are displayed as error bars).

Based on the fact that the majority of BBE‐like enzymes in bacteria and fungi oxidize alcohol groups of a variety of complex saccharide structures, an initial screening for enzymatic activity focused on mono‐, di‐, and polysaccharides (for a complete list of compounds, see Experimental procedures). This promptly revealed that *Pp*BBE1 oxidizes the two 1,4‐β–linked disaccharides cellobiose and lactose. A more detailed kinetic analysis by steady‐state kinetics yielded a higher apparent *k*_cat_ and lower apparent *K*_M_ for cellobiose, that is, 48 s^−1^ vs 28 s^−1^ and 0.6 mm vs 4.2 mm, respectively, indicating that the catalytic efficiency with cellobiose is 10 times higher than with lactose (*k*_cat_/*K*_M_ = 8 × 10^4^ m ^−1^·s^−1^ and 7 × 10^3^ m ^−1^·s^−1^ for cellobiose and lactose, respectively; Fig. 5). In order to identify the oxidation product, cellobiose was used in a scale‐up experiment to obtain sufficient amounts of product, which was subsequently analyzed by NMR spectroscopy (see Experimental procedures for details). The NMR spectra clearly demonstrate the formation of cellobionolactone, that is, the free anomeric center of cellobiose is oxidized to the corresponding lactone, which spontaneously hydrolyses to the ring‐open acid form (see Fig. S1). Finally, we determined the pH‐dependence of the reaction under steady‐state conditions. As shown in Fig. 6, the activity of *Pp*BBE1 reached a maximum at pH 5.8 (black squares).

### Presteady‐state experiments with cellobiose

To further evaluate the reductive and oxidative half‐reaction of the enzyme, we conducted presteady‐state experiments in the stopped‐flow apparatus (see Experimental procedures). First, the rate of reduction in *Pp*BBE1 was measured as a function of cellobiose concentration. The rate of reduction increased linearly with the cellobiose concentration and saturation was not observed up to 1 mm. At higher concentrations, the observed rate constants could not be extracted reliably due to the dead time restrictions of the instrument. Due to this limitation, we were unable to determine or fit a limiting rate of reduction and a dissociation constant for cellobiose. Therefore, we have used the determined bimolecular rate constant of 1.7 × 10^5^ m ^−1^·s^−1^ for further comparison and discussion. The rate of reoxidation of the reduced flavin by dioxygen was determined at a final oxygen concentration of 140 μm to 0.5 × 10^5^ m ^−1^·s^−1^ and is thus ca. 3 times slower than the rate of reduction.

### X‐ray crystallographic structure of *PpBBE1*

**Figure 7:**
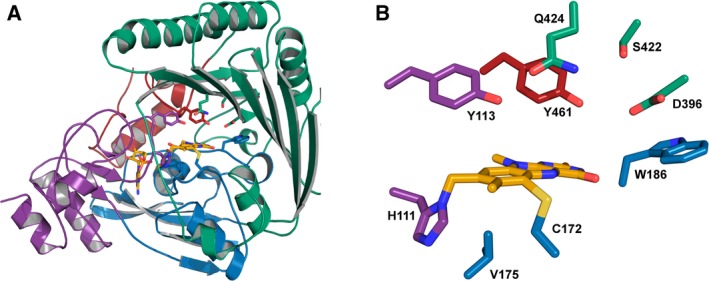
Overall crystal structure (A) and close‐up view of the active site (B) of *Pp* BBE1. (A) Structure of the *Pp* BBE1 as determined by X‐ray crystallography shown in cartoon representation, with the subdomain involved in substrate highlighted in green and the FAD‐binding domain in purple, blue, and red. The FAD cofactor (yellow) and the two amino acids responsible for its bicovalent attachment are shown as sticks (H111, purple and C172, blue). (B) Close‐up of the active site of *Pp* BBE1. The function of the amino acid residues shown were probed by site‐directed mutagenesis (see text for details).

In order to gain further insights into the structure–function relationships in the active site of *Pp*BBE1 and to develop a more detailed enzymatic reaction mechanism, we solved the three‐dimensional structure by X‐ray crystallography. Hanging drop vapor‐diffusion crystallization setups was used to obtain light yellow, tetragonal bipyramidal crystals of wild‐type protein as well as a protein variant (D396N) that was generated in the course of our site‐directed mutagenesis study (see site‐directed mutagenesis of putative active site residues). Crystals of the D396N variant generally diffracted to higher resolution and data generated from these crystals was, therefore, used for phasing of the structure. Including data up to a resolution of 2.6 Å, the structure was solved by molecular replacement using a homology model based on *At*BBE‐like 15 (PDB: 4UD8; 3) as template, confirming a protein fold, that had previously been shown for BBE‐like enzymes (Fig. 7) 2. An identical structure was then confirmed for the wild‐type data refined to 2.9 Å after phasing with the initial model of the variant. *Pp*BBE1 crystallized as a loose dimer in the asymmetric unit with a dimerization interface of no biological significance according to PISA analysis 21. In line with gel filtration experiments, *Pp*BBE1 is, therefore, expected to be functional as a monomer. Individual protomers also showed no substantial structural differences with a root‐mean‐square deviation of 0.15 Å for 404 aligned backbone Cα residues. A closer inspection of the active site revealed a relatively large active site with clear interpretable electron density of all active site residues. In addition, continuous electron density between the 8α‐ and C6‐positions of the flavin cofactor and residues H111 and C172, respectively, confirmed the bicovalent linkage of the flavin cofactor. Both wild‐type and the D396N structure featured a pronounced density at the expected site of substrate binding that could not be interpreted at the current resolution limits. Interestingly, the density projects toward the C4a atom of the flavin cofactor and might thereby explain the unexpectedly weak yellow coloration of the crystals. Further studies are needed to identify the source of this copurified ligand species.

**Figure 8:**
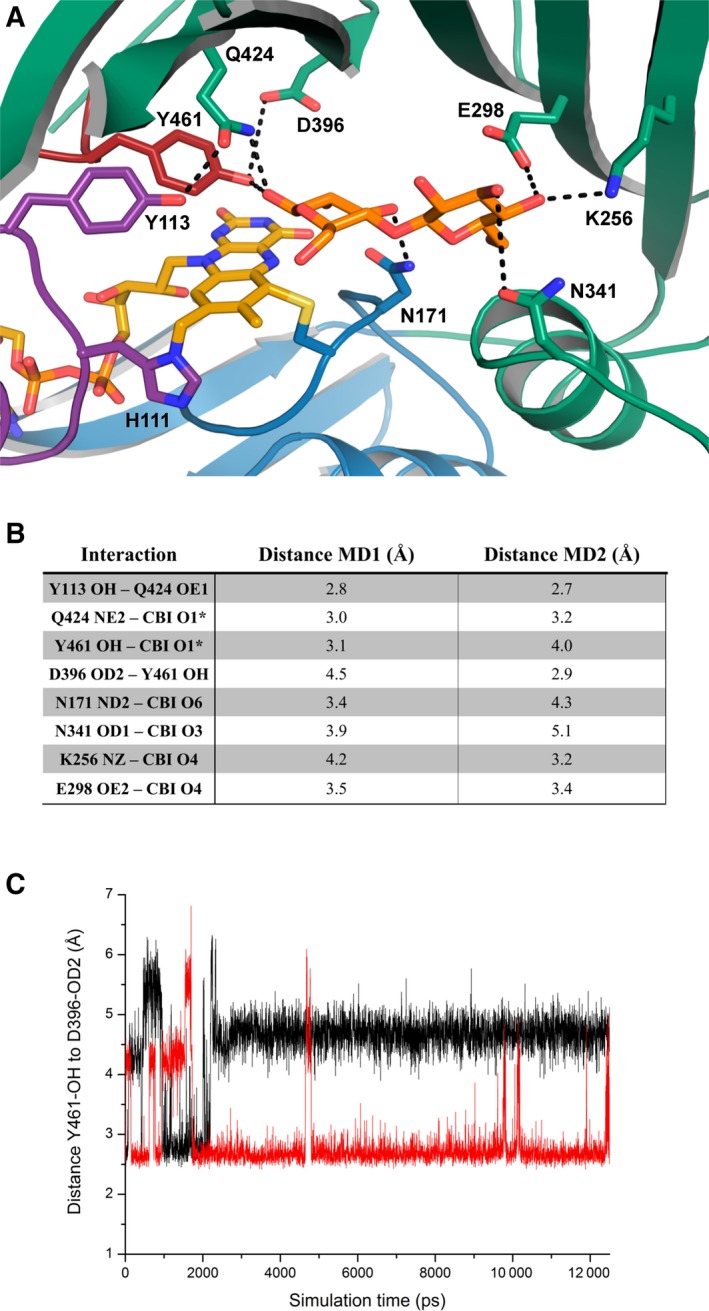
Results from molecular dynamic simulations performed after docking of cellobiose to the crystal structure of *Pp* BBE1. (A) Cellobiose‐binding mode, including hydrogen‐bonding interactions predicted by MD‐simulations (for color coding, see Fig. 6; cellobiose is shown as orange sticks). (B) Distances between possible interaction partners as predicted in two independent MD‐simulation runs (see black dashed lines in panel A). (C) Time‐dependent change in the distance between the two catalytic residues Y461 and D396 monitored for 12.5 ns.

The closer inspection of the active site revealed six residues with a potential role in substrate binding and activation (Fig. 8). Among these residues, two are likely to be required for substrate activation. D396 forms a hydrogen bond with Y461 and thereby may assist in the deprotonation of the tyrosine residue, which, in turn, could serve as catalytic base. Q424, which is in close vicinity to Y461 and D396, might also contribute to the activation of Y461, but could also play a role in substrate coordination or activation. The role of W186 and S422 is less obvious. Being able to interact with peptide backbone of L397 and the side chain of Y398, respectively, these two residues might be responsible for the correct positioning of D396 relative to Y461, but from the crystal structure, their importance is difficult to predict.

### Site‐directed mutagenesis of putative active site residues

In order to obtain more detailed information on the role of various active site residues, we initiated a site‐directed mutagenesis program. Thus, all six amino acid residues found in close vicinity of the flavin cofactor were replaced, that is, tyrosine 113 and 461 to phenylalanine (Y113F and Y461F), aspartic acid 396 to alanine and asparagine (D396A and D396N), glutamine 424 to alanine (Q424A), tryptophan 186 to phenylalanine (W186F), and serine 422 to alanine (S422A). In addition, the residue held responsible for controlling the oxygen reactivity on the *si*‐side of the isoalloxazine ring, that is, valine 175, was replaced by leucine (V175L; Fig. 8). As shown in the summary of kinetic parameters in Table 1, the replacement of Y461, D396, W186, S422, and Q424 significantly affected the catalytic rates whereas replacement of Y113 caused only marginal changes compared to the wild‐type enzyme. These results further support the hypothesis that D396 acts as an active site base and therefore determines the pH‐profile of the enzyme. This concept is also in agreement with the observation that replacement of D396 by an alanine resulted in a substantial shift of the pH‐optimum from ~ 5.8 to ~ 7.2 (Fig. 6, red spheres).

**Table 1:** Presteady‐state and steady‐state parameters determined for wild‐type *Pp*BBE1 and the protein variants. All measurements were performed in 50 mm HEPES buffer, pH 6, that is, near the pH optimum found for wild‐type *Pp*BBE1. In the presteady‐state experiments, no saturation could be observed up to 1 mm cellobiose, where the reaction was complete within the dead time of the instrument. Thus, a linear fit was applied in the range from 100 to 800/1000 μm in order to allow comparison with the variants. The slope obtained from this fit is presented as a bimolecular rate constant *k*_red_ (m ^−1^·s^−1^). The turnover rate (*k*_obs_) refers to the reaction rates determined under steady‐state conditions, using 1 mm cellobiose as substrate and 100 μm DCPIP as final electron acceptor

	*k*_red_ (m ^−1^·s^−1^)	*k*_red_ (%)	*k*_obs_ (s^−1^)	*k*_obs_ (%)
WT	1.7 × 10^5^	100	27 ± 0.70	100 ± 8
Y113F	9.7 × 10^4^	57	25 ± 0.50	91 ± 6
Y461F	n.d.	–	0.13 ± 0.20	0.50 ± 0.1
D396A	4.3 × 10^3^	2.5	0.91 ± 0.07	3.4 ± 0.2
D396N	1.4 × 10^3^	0.8	0.65 ± 0.05	2.4 ± 0.2
Q424A	n.d.	–	0.17 ± 0.02	0.60 ± 0.02
W186F	1.2 × 10^3^	0.7	0.47 ± 0.05	1.7 ± 0.1
S422A	7.0 × 10^3^	4.1	3.3 ± 0.05	13 ± 0.1

Previously, we established that the oxygen reactivity of the reduced flavin in the BBE‐like enzyme family is controlled by a single ‘gate keeper’ residue on the *si*‐side of the isoalloxazine ring. In the case of *Pp*BBE1, V175 is found in the pertinent ‘gate keeper’ position, and thus, the reduced enzyme is expected to rapidly react with dioxygen, as confirmed by the high rate of reoxidation, that is, 0.5 × 10^5^ m ^‐1^ s^‐1^. In accordance with our ‘gate keeper’ model, replacement of this valine by leucine diminished the rate of reoxidation to 25 m ^−1^·s^−1^, thus basically suppressing the reaction of the reduced flavin with dioxygen as previously found for other members of this enzyme family 3, 20, 22.

### Molecular docking and molecular dynamics simulations

As the substantial decrease in enzymatic activity of some variants could not be fully explained based on the crystal structure alone, molecular docking and subsequent molecular dynamics simulations with *Pp*BBE1 and cellobiose were performed. The docking experiment resulted in a reasonable binding of cellobiose to the active site of *Pp*BBE1, though some distances between the ligand and interacting residues were not in a favorable range. Therefore, two independent molecular dynamics simulations based on the previously obtained docking results were conducted (Fig. 8A).

The resulting average structures demonstrated a high flexibility of Y461, as the distance between D396 and the tyrosine as well as between the tyrosine and the anomeric hydroxyl group of cellobiose strongly differed in the two simulations (Fig. 8B,C). In one simulation, Y461 is in hydrogen‐bonding distance with D396, thus indicating the activation of tyrosine for catalysis, whereas the second simulation better represents the deprotonation of the anomeric hydroxyl group of the substrate molecule.

Furthermore, the significant decrease in enzymatic activity resulting from the exchange of Q424 by alanine can be better explained based on our *in silico* data. The amide nitrogen of Q424 directly points toward the C1* hydroxyl group of cellobiose and thus may have a role in stabilization of the catalytic transition state. In addition, the simulations indicated the involvement of several other amino acid residues in substrate binding: Q298 and K256 are both in hydrogen‐bonding distance to the C4 hydroxyl group of bound cellobiose (Fig. 8B) suggesting that they are involved in substrate recognition. Their suggested role in substrate binding also explains the higher *K*_M_ of lactose compared to cellobiose, as the C4 hydroxyl of lactose is an axial position and thus points away from these two amino acids.

### Generation and analysis of BBE‐deficient moss mutants

**Figure 9:**
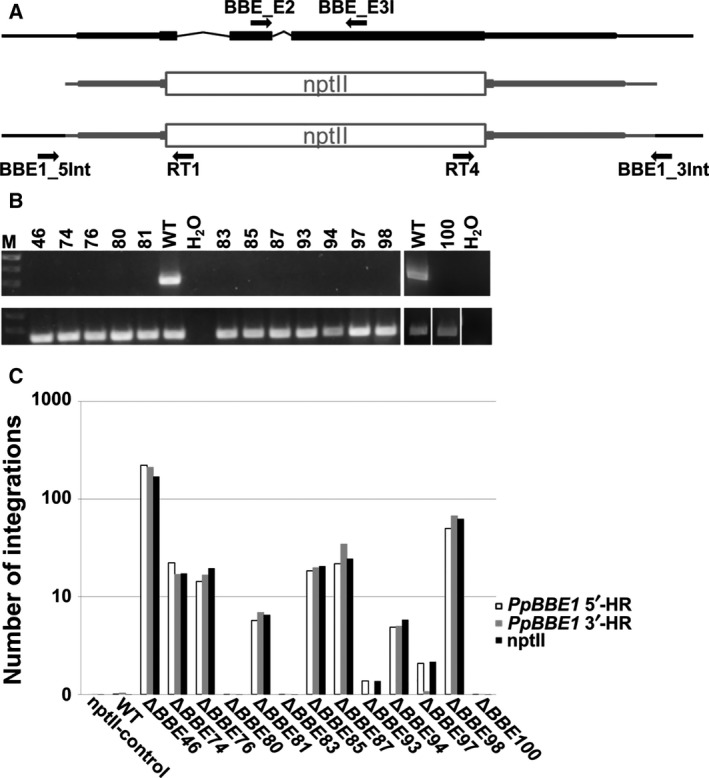
Molecular validation of Δ*PpBBE1* lines. (A) Schematic representation of the genomic locus (top), the *PpBBE1*‐targeting construct in which most parts of the *PpBBE1*‐coding sequence is replaced by the nptII selection marker (middle) and the genomic locus in Δ*PpBBE1* (bottom). The targeting construct is depicted in gray color, the native genomic locus in black color. Exons are represented by boxes, UTRs as slim boxes and introns or the intergenic region 5′ and 3′ of *PpBBE1* as lines. Primers used for screening of correct 5′‐ and 3′‐integration of the targeting construct into the genome are BBE_5Int and RT1 or RT4 and BBE_3Int, respectively, for RT‐PCR BBE_E1 and BBE_E2I; (B) Loss of *PpBBE1* transcript is validated via RT‐PCR in 13 Δ*PpBBE1* lines (Δ*PpBBE1*–*46, 74, 76, 80, 81, 83, 85, 87, 93, 94, 97, 98, 100*), in WT the product size using BBE_E1 and BBE_E2I is 546 bp, as negative control water (H_2_O) was included (top). Control for successful cDNA synthesis is shown for all lines (bottom). (C) Quantification of targeting construct integration numbers in the validated Δ*PpBBE1* lines with qPCR using three different primer pairs, amplifying a part of the 5′‐ and 3′‐homologous region (*PpBBE1* 5′HR or *PpBBE1* 3′HR) or the coding sequence of the nptII selection marker (nptII). As controls a line with a single integration of the nptII selection marker (nptII‐control) and WT were used.

To analyze the function of *Pp*BBE1 *in vivo,* we generated moss Δ*PpBBE1* knockout mutants deleting the coding sequence almost completely. A 3.6 kb long fragment was amplified from genomic DNA and the central part of this fragment comprising nearly the whole‐coding sequence leaving only 38 bp of exons 1 and 37 bp of exon 3 was replaced by the nptII selection cassette (Fig. 9A). After poly(ethylene glycol)‐mediated protoplast transformation (done in six independent approaches), antibiotic selection, and regeneration 23, 915 surviving colonies were picked. In an initial screening of 28 colonies by leaflet PCR 24, 12 putative Δ*PpBBE1* lines were identified and validated by RT‐PCR (Fig. 9B). Flow cytometry analysis 25 confirmed that these lines were haploid. Subsequently, the copy number of the targeting construct was analyzed with a qPCR‐based method on genomic DNA (Fig. 9C; 26). For all further experiments, three Δ*PpBBE1* single integration lines (#80, #83 and #100) were used.

**Figure 10:**
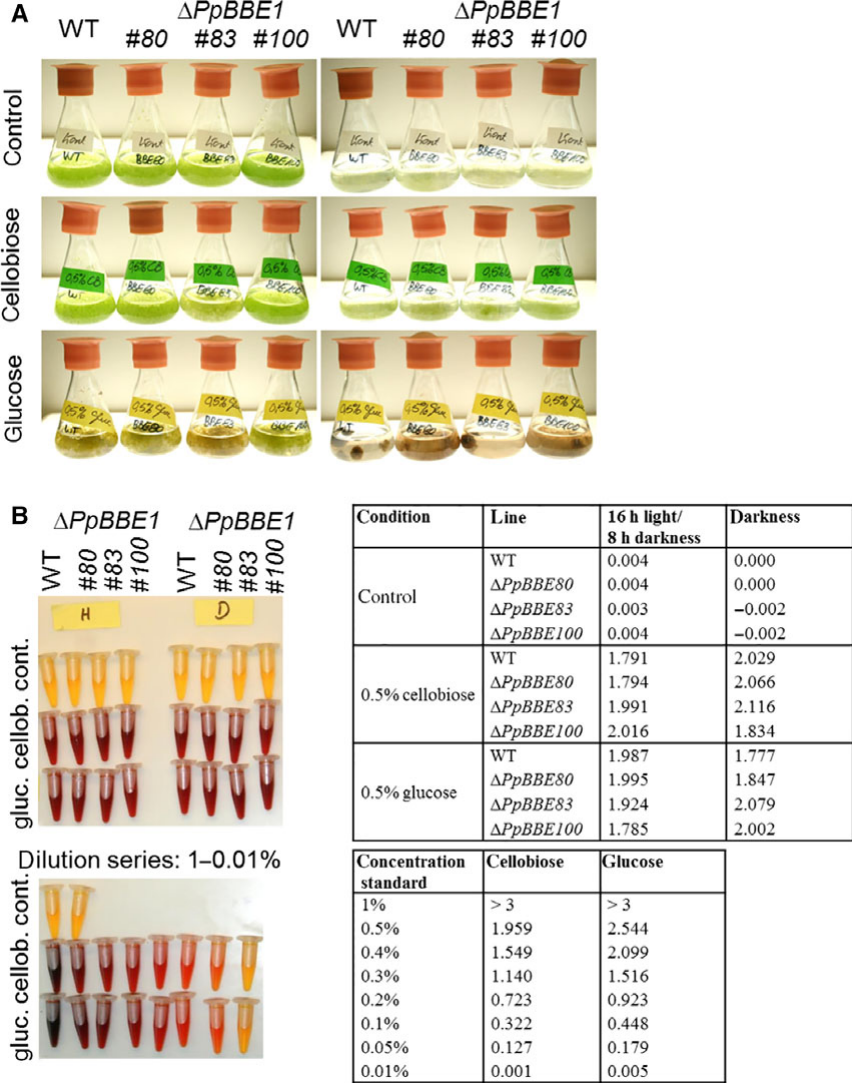
Protonema cultures of WT and Δ*PpBBE1* plants grown in liquid medium without or with 0.5% cellobiose or 0.5% glucose as a source of carbohydrates and assay of sugars in the media after 21 days of growth. (A) Flasks with protonema cultures of WT or Δ*PpBBE1* lines grown under standard growth conditions with a 16 h light and 8 h darkness regime (left panel) or in complete darkness (right panel). (B) Determination of glucose and cellobiose in the culture media compared with a dilution series of the sugars in the control growth medium with the DNS assay resulting in a concentration‐dependent coloring from dark red (high concentration of reducing sugars) to yellow (no reducing sugars detected) in the left panel. The absorbance of the samples and dilution series shown in the left panels measured at 575 nm is shown in the tables.

**Figure 11:**
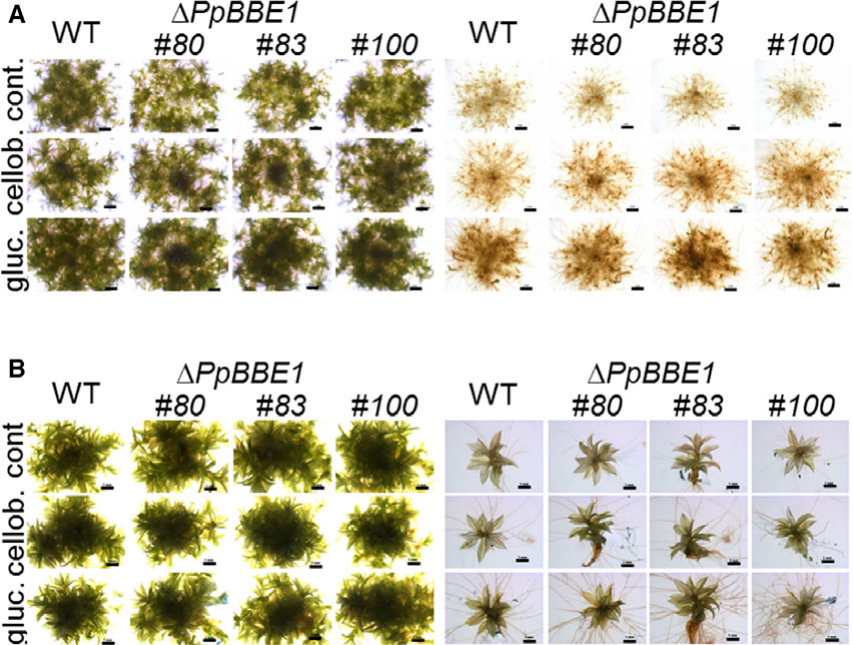
Growth of WT and Δ*PpBBE1* plants on solid media without or with 0.5% cellobiose or 0.5% glucose as a source of carbohydrates. (A) Protonema spot inocula grown on solid media for 6 weeks. Left panel: 16 h light and 8 h dark regime, right panel: 2 weeks of growth in a 16 h light and 8 h dark regime before transfer to darkness for 4 weeks. Size bars: 1 mm; (B) Single gametophores grown for 4 weeks. Left panel: 16 h light and 8 h dark regime, right panel: darkness. Size bars: 2 mm. Cont.: control (medium without supplements); cellob.: medium containing 0.5% cellobiose; gluc.: media containing 0.5% glucose.

Growth and morphology of Δ*PpBBE1* plants were unchanged compared to WT throughout the whole life cycle from protoplast regeneration to sporophyte development. Also, upon salt stress, neither gametophores on solid plates nor protonema in liquid culture showed a phenotype differing from WT. In order to study the effect of Δ*PpBBE1* on the degradation of the carbohydrate cellobiose, the plants were grown on media supplemented with either 0.5% cellobiose or 0.5% glucose, which is known to delay differentiation by prolongation of juvenile stages 27 and also promotes growth of *Physcomitrella* cultures in low light conditions 28. Growth and development of Δ*PpBBE1* lines were studied with and without supplementation of carbohydrates in standard growth conditions (16 h light, 8 h darkness) or darkness. Therefore, protonema cultures were grown in liquid medium (Fig. 10A) and protonema spot inocula (Fig. 11A) or single gametophores (Fig. 11B) were used as starting material on solid medium. In all conditions, no difference between Δ*PpBBE1* and WT was detected regarding growth rate and development. The addition of cellobiose to cultures grown in the dark led to an intermediate phenotype between the control without a carbohydrate source and media supplemented with glucose, but again no significant difference between the WT and the knockout strain could be observed.

In addition to the phenotypical analysis, qualitative and quantitative analysis of the glucose and cellobiose content in the supernatant of the liquid cultures was performed. After 3 weeks of incubation, the presence of reducing sugars was assayed with the Benedict's test, before conducting a quantitative analysis using the 3,5‐dinitrosalicylic acid (DNS) assay (Fig. 10B) and glucose test strips (for details the Experimental procedures). As before, no differences between Δ*PpBBE1* and WT cultures were observed. Similarly, cultures that were grown on plates for 4–6 weeks were analyzed in terms of the carbohydrate content in the solid media using congo red staining. Again, this analysis did not reveal any difference between Δ*PpBBE1* and WT.

## Discussion

**Scheme 1:**
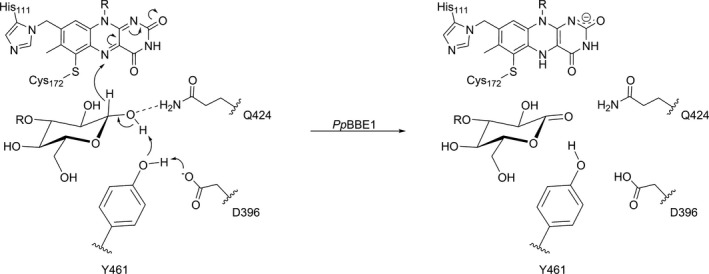
Proposed reaction mechanism for the oxidation of cellobiose to the corresponding cellobionolactone catalyzed by *Pp* BBE1. D396 is expected to deprotonate the side chain ‐OH of Tyr461, which thereby gets activated to act as catalytic base. By abstracting the proton from the anomeric hydroxyl group (reducing end), lactone formation is triggered and a hydride in transferred from the anomeric carbon to the N5‐position of the FAD cofactor. The latter thereby gets reduced and is regenerated upon reoxidation by molecular oxygen.

The successful expression of gene #1 (*Pp*3c12_2640V3.1) in *K. paffii* led to the production of suitable amounts of *Pp*BBE1 and enabled us to carry out the biochemical and structural characterization of the enzyme. In the course of our studies, we identified the disaccharide cellobiose as the best substrate, which is oxidized at the free anomeric center to the corresponding cellobionolactone and spontaneously hydrolyses to cellobionate. The rate of cellobiose oxidation is very fast and exceeds the rate observed for many other flavin‐dependent oxidases 29, 30, 31, 32. In fact, the observed bimolecular rate constant already at rather low substrate concentrations is greater than the rate of reoxidation of the reduced flavin by dioxygen, which was also observed for other enzymes belonging to the BBE‐like protein family. Thus, *Pp*BBE1 clearly harbors a very efficient active site for the oxidation of cellobiose. In order to understand the catalytic contributions of the amino acids in the active site, we have generated a set of seven variants and determined the relevant kinetic parameters (Table 1). In conjunction with the crystal structure of *Pp*BBE1, we are now in a position to propose an enzymatic reaction mechanism for the oxidation of cellobiose. As shown in Scheme 1, we propose that the side chain carboxylate group of D396 deprotonates the neighboring side chain of Y461, which in turn abstracts a proton from the anomeric hydroxyl group of the substrate leading to the transfer of a hydride to the N5‐position of the flavin. The relevance of this catalytic diade is in accordance with the observed effects on the rate of substrate oxidation (Table 1) and the changes in the pH‐profile found for the D396A variant (Fig. 6). The positioning of the anomeric center in the vicinity of the side chain of Y461 and the N5‐position of the flavin was confirmed by substrate docking to our crystallographic structure of the protein. In addition, the characterization of the W186F and the S422A variant also revealed that these residues play an important role in organizing the active site, probably by ensuring the correct positioning of the catalytic base D396. This view is supported by the structural network established by these residues, which apparently stabilize the structural topology by interacting with the backbone carbonyl of L397 (W186) and the hydroxyl group of Y398 (S422 via a water molecule), respectively. Q424, on the other hand, may form a hydrogen bond with Y113, but in view of the total loss of enzymatic activity in the Q424A variant, direct stabilization of the transition state seems to be the primary function of this amino acid side chain. This finding is in line with the observation that, according to the crystal structure, Q424 forms only one hydrogen bond, whereas a possible second interaction with the hydroxyl group at the anomeric center of cellobiose is revealed in our docking simulations.

As mentioned in the introduction, we were particularly curious to identify a substrate (or a group of substrates) for *Pp*BBE1 because this would provide information on the ‘primordial’ function of the BBE‐like enzyme family in plants. In this context, the identification of cellobiose and lactose as substrates of *Pp*BBE1 is very intriguing since most members of the BBE‐like enzyme family in bacteria and fungi oxidize hydroxyl groups of various carbohydrate substrates, for example, aclacinomycin oxidoreductase from *Streptomyces* species 33 and GOOX from the rot fungus *Acremonium strictum* 18. Thus, it is apparent that this activity was already present in early land plants represented by mosses and constituted the starting point for the evolution of BBE‐like enzymes in vascular plants, where members of the family are engaged in the oxidation of cyanohydrines and monolignols, as described for *A. thaliana* 3, 4. The unusual oxidative formation of the berberine bridge in benzylisoquinoline biosynthesis and the cyclization of the monoterpene moiety of cannabigerolic acid to Δ^1^‐tetrahydrocannabinolic acid (THCA), catalyzed by *Ec*BBE and THCA synthase from *Cannabis sativa*, respectively, have clearly taken this enzyme family to an unprecedented new level of catalytic sophistication and reflect the diverse array of secondary metabolites found in several plant lineages. On the other hand, the observation that some plants possess BBE‐like enzymes with glucose oxidase activity, such as nectarin V from tobacco 7 indicates that the ability to oxidize carbohydrates was also conserved in plants. These observations are in tune with the current concept that biosynthetic pathways have evolved in part by gene duplication and subsequent neofunctionalization of one of the genes to support novel enzymatic modifications of metabolites leading to the enormous variety of compounds found in plants 9, 34. The increasing number of genes encoding members of the BBE‐like enzyme family during plant evolution as well as distinct changes in the active site composition suggests that this process was relevant for the evolutionary diversification of this enzyme family in the plant kingdom 2, 3.

Our *in vitro* findings are also in good accordance with the *in vivo* data obtained from the characterization of a WT and a *PpBBE1*‐knockout strain. The expression of *PpBBE1* was high in defined developmental stages, namely chloronema and protoplasts, which originate from protonema tissue. The chloronema harbors high photosynthetic activity characterized by a high number of chloroplasts of large size. On the other hand, caulonema cells possess fewer and smaller chloroplasts that are induced later by auxin and allow spreading of the colony by fast, radial growth relying on the availability of energy and carbon supply 28. The expression pattern of *PpBBE1* reflects the differences in the two protonema tissue types, being highly expressed in chloronema, but not detectable in caulonema. This finding nicely correlates with the recently performed transcriptome analysis of protoplasts 35 as well as of chloronema cells 10, 11 that have shown a significant upregulation of carbohydrate and energy metabolism in these early stages of the plant's life cycle. Our hypothesis that *Pp*BBE is involved in carbohydrate degradation is further supported by the fact that also fungi use cellobiose for energy production, which is initially metabolized extracellularly to cellobionic acid and then further degraded by cellobionic acid phosphorylases after transport into the cytoplasm. The resulting products, gluconate and glucose‐1‐phosphate, are then converted to 6‐phosphogluconate and glucose‐6‐phosphate before entering the pentose phosphate pathway and glycolysis, respectively 36. Even though this catabolic pathway has never been reported for plants, it is possible that *P. patens* has a similar set of enzymes allowing it to most efficiently use cellulose for energy production 37.

Similarly, *PpBBE1* expression was detected in the green parts of the gametophore but not the rhizoids, root‐like structures with a similar morphology as caulonema filaments. On transition of the gametophyte to the sporophyte, a transcriptomic switch occurs regarding carbohydrate metabolism and photosynthesis, as the gametophyte has a primary role in photosynthesis, resource acquisition, and long‐term maintenance of the plant, while the sporophyte predominantly relies on the gametophyte for both carbon and energy 10, 38, 39. This switch is also reflected by the expression of *PpBBE1*, which is very low or not detectable throughout the sporophyte development to the mature spore.

Interestingly, the knockout of *PpBBE1* showed no apparent impact on plant morphology and development throughout the whole life cycle in all conditions tested in this study. Even upon growth in darkness, when assimilation via photosynthesis does not take place and supplementation of the media with cellobiose or glucose as carbohydrate source is required for heterotrophic growth, no differences were detected comparing Δ*PpBBE1* and WT plants. The effect of glucose and sucrose on *Physcomitrella* grown in the dark leading to increased caulonema formation has been well studied 28, 40. In our study, the phenotype of cultures grown in cellobiose containing growth media is an intermediate between controls without a carbohydrate source and glucose supplemented cultures although, also here, no difference between the WT and the knockout strains was detected. These findings indicate that cellobiose is metabolized like other carbohydrates such as glucose, used as positive control, or sucrose. As upon heterotrophic growth, not only the phenotype and development of Δ*PpBBE1* compared to WT plants but also the concentration of cellobiose or glucose in culture supernatants was unchanged, we conclude that *Pp*BBE1, for which we identified cellobiose as best substrate, is not the only enzyme active in cellobiose metabolism in *Physcomitrella*. Therefore, we propose that the deletion of *PpBBE1* is compensated by the action of other enzymes involved in cellobiose turnover.

## Experimental procedures

### Materials

All chemicals and media ingredients were purchased from Sigma‐Aldrich (St. Louis, MO, USA), Roth (Karlsruhe, Germany), Merck (Darmstadt, Germany), Fluka (Buchs, Switzerland) or Becton, Dickinson and Company (Franklin, Lakes, NJ, USA) and were of the highest grade commercially available.

All restriction enzymes used were ordered form Thermo Scientific/Fermentas (St. Leon‐Rot, Germany) or New England Biolabs (Ipswich, MA, USA), while the Ni‐sepharose column material was obtained from GE Healthcare (Chalfont St. Giles, UK).

### Cloning and expression

For heterologous expression of the *PpBBE*‐gene in *K. phaffii*, the respective gene was purchased from Thermo Scientific. On ordering, the native sequence was codon optimized for *K. phaffii* and nucleotides coding for an octahistidine tag were fused to the 3′‐end of the gene.

After subcloning into the *Escherichia coli* pJET vector, PCR amplification was performed in order to remove the native signal sequence identified using SignalP. This PCR product was then cloned into the *K. phaffii* vector pPICZα, to enable secretion of *Pp*BBE1 in the non‐native host. Having linearized the DNA using SacI, electroporation following the instructions provided by the EasySelect™ ExpressionKit (Invitrogen, Waltham, MA, USA) was applied to transform KM71H cells with the gene of interest. These cells were also transformed with pPICK‐PDI plasmid DNA to be able to coexpress of the protein disulfide isomerase from *Saccharomyces cerevisiae*.

Having identified stable and well‐expressing clones in small‐scale screenings, following the instructions given by 41, large‐scale expression in a BBI CT5‐2 fermenter (Sartorius, Göttingen, Germany) was performed as described by 42. After 96 h of MeOH feed (induction time), the pH was set to 8, and cells and medium were separated by centrifugation (3500 ***g*** for 20 min). The cells were discarded and the supernatant was incubated with 30 mL of Ni‐Sepharose™ (GE Healthcare) Fast Flow column material, which had been equilibrated with binding buffer (50 mm NaH_2_PO_4_, 150 mm NaCl pH 8) prior to use. To ensure binding of all tagged protein, 1 h of stirring at 4 °C was required, before the column material was filtered and packed into an empty column (XK 26). Having washed with ~ 150 mL of wash buffer (50 mm NaH_2_PO_4_, 150 mm NaCl, 20 mm imidazole pH 8) to remove all the unspecifically bound proteins, *Pp*BBE1 was eluted with elution buffer (50 mm NaH_2_PO_4_, 150 mm NaCl, 300 mm imidazole pH 8). SDS/PAGE analysis was then used to determine the quality of the purification, before pooling the fractions with the highest protein content and lowest amount of impurities (typical yields: 5–7 mg·L^−1^ of culture; ~ 98% purity). Subsequently, the protein solution was concentrated and the buffer was exchanged to 50 mm HEPES buffer pH 6 using Centripreps® 30 kDa MWCO (Millipore Merck, Burlington, MA, USA).

### Protein denaturation

To be able to determine the protein concentration based on the characteristic absorption of FAD at about 450 nm, a spectrum of the native as well as of denatured *Pp*BBE1 was recorded. Assuming that the extinction coefficient of the flavin species found in denatured *Pp*BBE1 is equal to the one of 6‐*S*‐cysteinyl FMN, an extinction coefficient of 9800 m ^−1^·cm^−1^ could be calculated for native *Pp*BBE1 at 443 nm.

### Photoreduction

Photoreduction was performed as described by Massey *et al*. 43. Under anaerobic conditions, quartz cuvettes were filled with 20 μm enzyme solution, which also contained 5 mm EDTA as photosubstrate as well as 5 μm 5‐deaza‐FMN. Having recorded a UV/VIS spectrum of the native, oxidized enzyme, the sample was irradiated and further spectra were recorded at different time points. In order to avoid heat denaturation of the protein, the cuvettes were constantly cooled to 15 °C.

### Determination of the redox potential

The redox potential of the flavin cofactor was determined using the dye‐equilibrium method based on the xanthine/xanthine oxidase system first reported by Massey 19. Two separate solutions, one containing 500 μm xanthin, 5 μm methyl viologen, and 30 μm *Pp*BBE1 and a second one with xanthin oxidase (about 5 ng) and toluyene blue (*A*_647_ = 0.3; E°: +115 mV) were prepared in 50 mm HEPES buffer pH 7. By mixing these solutions in a stopped flow device (SF‐61DX2; Hi‐Tech, TgK Scientific Limited, Bradford‐on‐Avon, UK), placed in an anaerobic glove box, the reaction was started. To monitor the reduction in the enzyme and the dye, 300 absorption spectra (350–700 nm) were recorded with a KinetaScan T diode array detector (MG‐6560) from Hi‐tech, within 50 min. The log ([ox]/[red]) of the enzyme was plotted against the log ([ox]/[red]) of the dye allowing calculation of the redox potential as described by Minnaert 44.

### Substrate screening

Since the physiological role of *Pp*BBE1 was not known, we performed a screening in 96‐well plates using 1.5 mm 2,6‐dichloroindophenol in 50 mM HEPES buffer pH 8, containing 1 mm of the following substrate, and 1 μm of purified enzyme: d‐saccharose, d‐fructose, d‐glucose, d‐galactose, d‐mannose, L‐arabinose, d‐arabinose, d‐sorbitol, d‐mannitol, starch, d‐maltose, d‐lyxose, l‐fucose, d‐lactulose, d‐ribose, d‐xylose, d‐trehalose, cellulose, hydroxyethylcellulose, lignin, amylose, ribitol (adonitol), d‐lactose, d‐cellobiose, and cellotriose. The plates were incubated at room temperature and decolorization of the DCPIP solution was analyzed as a function of time.

### pH‐optimum

Having identified two well‐accepted substrates for *Pp*BBE1, the pH‐optimum was determined in order to be able to study the enzyme characteristic kinetic properties at the optimal pH. Therefore, different buffers in the pH‐range of 4–9 were prepared and substrate conversion was analyzed for the wild‐type as well as for the variant enzymes. Since both substrate and product do not have any characteristic spectral properties, a coupled assay with DCPIP (100 μm final concentration) was established. By recording the change in absorption at 600 nm, substrate conversion could be monitored and the pH‐optimum could be determined. All measurements were performed with constant substrate concentration (1 mm cellobiose) and an enzyme concentration of 10 nm for the wild‐type and 200 nm for the variant enzymes, respectively.

### Steady‐state kinetics

Steady‐state kinetics of *Pp*BBE1 were measured with cellobiose and lactose for the wild‐type enzyme and all active site variants were subsequently only measured with the more efficiently converted cellobiose substrate. A coupled assay with DCPIP (100 μm final concentration in 50 mm HEPES pH 6) was used to determine the initial rates observed upon altering the substrate concentration (cellobiose: 50–1500 μm; lactose: 250–8000 μm), while keeping the enzyme concentration constant. As for the analysis of the pH‐optimum, final enzyme concentrations of 10 nm for the wild‐type and the Y113F variant and of 200 nm for the remaining variants were used. All reactions were monitored at 600 nm for 120 s and were performed in triplicates.

By plotting the extracted initial velocities vs the respective substrate concentration, *v*_max_ as well as apparent *K*_M_ values could be determined.

Due to the low reactivity of protein variants, no full Michaelis–Menten curve was recorded. Instead, conversion rates at 1 mm cellobiose were used for an internal comparison and measured in triplicates.

### Presteady‐state analysis

For analysis of the reductive and the oxidative half‐reaction, a stopped‐flow device from Hi‐Tech (SF‐61DX2) installed in a glove box (Belle Technology, Cambridge, UK) was used. In both cases, time‐dependent spectral changes of the flavin absorption were monitored either with a KinetaScanT diode array detector (MG‐6560) or a photomultiplier (PM‐61s). Data analysis was then performed with the Kinetic Studio Software (TgK Scientific Limited) by fitting the data points recorded at 443 nm.

To study the reductive half‐reaction, observed rate constants for flavin reduction at six different substrate concentrations (100–1000 μm) were determined in 50 mm HEPES pH 6 (at higher substrate concentrations, the reaction was too fast to reliably fit the data to extract rate constants). By plotting *k*_obs_ vs the corresponding substrate concentration, a linear increase in reaction rate constants could be observed, making it impossible to fit a limiting reductive rate. In order to allow comparison with the active site variants, a linear fit was applied and the slopes were used for data interpretation.

The oxidative half‐reaction was studied by following the reoxidation of the flavin upon mixing enzyme, reduced with 1.2 eq of substrate, with air‐saturated buffer (20 °C). To obtain bimolecular rate constants, the rates determined in this experiment were divided by the amount of oxygen dissolved in the buffer (final concentration: 140 μm).

### Site‐directed mutagenesis

In order to study the reaction mechanism in more detail, active site variants were produced. All constructs were generated from pPICZα‐*Pp*BBE1 wild‐type expression strain applying polymerase chain reaction‐based mutagenesis. The amino acid exchanges were introduced with the forward and reverse primers that both carried the desired mutations in the nucleotide sequence (for primer sequences, see Table 2).

**Table 2:** Sequences of the primers used for site‐directed mutagenesis, with the codon triplet carring the mutation shown in bold

Variant	Type	Sequence
Y113F	Fwd.	5′‐GTGGTCACTCT**TTC**GAGGATTACTC‐3′
	Rev.	5′‐GAGTAATCCTC**GAA**AGAGTGACCAC‐3′
V175L	Fwd.	5′‐CTGTCCAACT**CTG**GGTATTGCTGGTCATG‐3′
	Rev.	5′‐CAATACC**CAG**AGTTGGACAGTTACCAGCTG‐3′
W186F	Fwd.	5′‐GGAGGTGGT**TTC**GGTTTTTCATC‐3′
	Rev.	5′‐ GATGAAAAACC**GAA**ACCACCTCC ‐3′
D396N	Fwd.	5′‐GCTTACTTCATCTAC**AAC**TTGTACGG‐3′
	Rev.	5′‐CCGTACAA**GTT**GTAGATGAAGTAAGC‐3′
D396A	Fwd.	5′‐GCTTACTTCATCTAC**GCT**TTGTACGG‐3′
	Rev.	5′‐CCGTACAA**AGC**GTAGATGAAGTAAGC‐3′
S422A	Fwd.	5′‐CTTGTAC**GCT**ATCCAGATGGTTGCTTCCTG‐3′
	Rev.	5′‐CCTGGAT**AGC**GTACAAGGAATTTCTGTGGATG‐3′
Q424A	Fwd.	5′‐GTACTCTATC**GCT**ATGGTTGCTTC‐3′
	Rev.	5′‐GAAGCAACCAT**AGC**GATAGAGTAC‐3′
Y461F	Fwd.	5′‐GTCAGGCT**TTC**CAGAACTACATCG‐3′
	Rev.	5′‐CGATGTAGTTCTG**GAA**AGCCTGAC‐3′

After successful cloning and transformation of the mutant strains, expression and purification of the *Pp*BBE1 variants were performed as described for the wild‐type protein.

### NMR analysis of the oxidation product

For NMR product analysis, medium‐scale turnover reactions were performed, using 3 mL of 10 mm cellobiose solution (in ddH_2_O) and 200 nm enzyme. After 3 h of incubation at 25 °C, full conversion of the substrate was confirmed by thin layer chromatography [MeCN : H_2_O (85 : 15)]. The samples were then dried in an ISS110 SpeedVac® System (Thermo Scientific, Waltham, MA, USA) and subsequently redissolved in 750 μL of D_2_O. The last two steps were repeated twice, before NMR analysis was conducted. A Varian (Agilent, Santa Clara, California, USA) INOVA 500‐MHz NMR spectrometer (Agilent Technologies) and the VNMRJ 2.2D software were used for all measurements. ^1^H‐NMR spectra (499.98 MHz) were measured on a 5 mm indirect detection PFG‐probe, while a 5 mm dual direct detection probe with z‐gradients was used for ^13^C‐NMR spectra (125.71 MHz). HSQC and HMBC spectra were measured with 16 scans per increment and adiabatic carbon 180° pulses.

### Crystallization and data collection

Protein crystals were grown in hanging drop vapor diffusion setups using EasyXtal® 15‐Well Tools (Qiagen, Hilden, Germany) and the corresponding crystallization supports (Qiagen). Protein solution (200 μm in 50 mm HEPES buffer pH 6) and crystallization reagent (0.1 m Mg(OOCH)_2_, 13/14% poly(ethylene glycol) 3350 for wild‐type and variant, respectively) were mixed in 2 : 1 or 1.5 : 1 ratio and incubated at 20 °C. After overnight equilibration, streak seeding with crystals from the initial hit was performed. Ten to 14 days later, bipyramidal crystals appeared, which reached their final size after 3 weeks. For cryoprotecting crystals when flash freezing them in liquid nitrogen, 3 μL of 0.1 m Mg(OOCH)_2_, 13/14% poly(ethylene glycol) 3350, and 40% glycerol were added to the drop containing the *Pp*BBE1 crystals a few minutes prior to freezing. Crystals were stored in liquid nitrogen and data were collected at 100 K at beamline P11 of the German Electron Synchrotron (DESY, Hamburg, Germany).

### Data processing and structure elucidation

Data from the tetragonal bipyramidal crystals were processed with the XDS package 45. A unique molecular replacement solution was obtained in space group P*4*_*1*_ *2*_*1*_ *2* in Phenix Phaser 46, using a homology model generated on the SWISS‐MODEL sever 47, 48, 49 based on the crystal structure of *At*BBE‐like 15 (pdb code: 4UD8; 3) as template. The initial solution was refined in cycles of maximum‐likelihood least‐squares refinement of models modified with Coot 50 using σ_A_‐weighted 2m*F*_o_ − D*F*_c_ and *F*_o_ − *F*_c_ electron density maps and including an initial simulated annealing (torsion) step. Details of data collection, processing, and refinement are summarized in Table 3.

**Table 3:** Data collection and refinement statistics

	Wild‐type *Pp*BBE1 (pdb: 6EO4)	*Pp*BBE1 D396N (pdb: 6EO5)
Data collection		
Space group	P*4*_*1*_ *2*_*1*_ *2*	P*4*_*1*_ *2*_*1*_ *2*
Cell dimensions		
*a*,* b*,* c* (Å)	149.2/149.2/205.6	148.7/148.7/204.8
α, β, γ (°)	90/90/90	90/90/90
Wavelength (Å)	1.009	1.009
Resolution (Å)	48.6–2.9 (3.0–2.9)^a^	48.4–2.6 (2.7–2.6)
*R*_meas_	0.27 (2.45)	0.173 (2.16)
*I*/σ*I*	13.4 (1.6)	12.1 (1.2)
CC(1/2) (%)	99.8 (66.2)	99.7 (65.8)
Completeness (%)	99.9 (100)	99.9 (99.8)
Redundancy	25.9 (26.5)	14.7 (14.8)
Refinement		
Resolution (Å)	48.6–2.9	48.4–2.6
No. reflections	52 047	70 960
*R*_work_/*R*_free_	18.2/20.9	18.3/21.0
No. atoms		
Protein	7158	7158
Ligands	134	134
Solvent	10	131
*B*‐factors		
Protein	61.0	63.4
Ligand/ion	66.5	68.6
Solvent	50.6	58.6
R.m.s. deviation		
Bond lengths (Å)	0.009	0.006
Bond angles (°)	1.053	0.884

a Values in parentheses are for highest resolution shell.

### Molecular docking

The crystal structure of *Pp*BBE1 wild‐type (WT; PDB: 6EO4) in complex with the bicovalently linked FAD cofactor was used as a basis for substrate‐docking experiments. All molecular docking experiments and subsequent MD simulations were performed with the YASARA Structure suite (YASARA structure version 17.3.30 (Yasara Biosciences 51). Crystal waters were removed from the structure prior to the docking experiment. Molecular docking was conducted utilizing the Autodock Vina Plugin 52 of the Yasara Structure suite. Docking was performed with the rigid receptor while the ligand was flexible with a docking cell lining the active site cavity of *Pp*BBE1. The ligand chosen for the docking experiments was the previously identified substrate of *Pp*BBE1, cellobiose. Two hundred and fifty docking runs were performed with an RMSD cutoff of 1 Å. The resulting docking poses were inspected visually.

### Molecular dynamics simulation

The enzyme complex consisting of *Pp*BBE1, the bicovalently linked FAD cofactor and the previously docked substrate cellobiose were used as the starting structure for molecular dynamics (MD) simulations. MD simulations and analyses were performed using the YASARA Structure suite, version 17.3.30 (YASARA Biosciences 51). A periodic simulation cell that enveloped the whole enzyme with an additional 5 Å margin in each dimension was used with explicit solvent. The AMBER14 force field 53 was applied and long‐range electrostatic potentials were calculated using the Particle Mesh Ewald (PME) method and a cutoff of 7.864 Å 54, 55. AutoSMILES utility was used to attribute force field parameters to *Pp*BBE1 and FAD 56. The hydrogen‐bonding network optimization was carried out using the method of Hooft and coworkers 57, and p*K*_a_ values at pH 6 were assigned 58. The simulation cell was filled with water containing 0.9% of NaCl (density of 0.993 g·mL^−1^). Solvent was relaxed and the system was subsequently energy minimized using steepest descent minimization to remove conformational stress, followed by a simulated annealing minimization to convergence (< 0.05 kJ·mol^−1^ per 200 steps). Integration time steps were 1.33 and 4 fs for intra‐ and intermolecular forces, respectively. Subsequently, MD simulations at 310 K were performed, whereby integration time steps for intramolecular and intermolecular forces of 1.25 fs and 2.5 fs were applied, respectively. MD simulations were carried out over a time span of 12.5 ns. A restrain was added to the force field to maintain a catalytically competent complex at the active site. A restraining spring with a force of 100 SFC was applied combined with a distance restrain of 3 Å between the catalytically relevant hydrogen (H1*) of the substrate and the N5 of FAD. Additionally, an angle of 105° was set between N10‐N5 and the previously mentioned hydrogen atom (H1*) of the substrate. The restraints were set following the findings of defining general distances and angles for catalysis‐competent complexes of flavoenzymes and their respective substrates 59. Analysis of MD simulation results included measuring general distance traces and hydrogen‐bonding events of cellobiose with predefined amino acid side chains of *Pp*BBE1 that determine the structure of the active site.

### Secretome analysis

For secretome analysis, the liquid culture supernatant of a *P. patens* line secreting human recombinant Erythropoietin (IMSC40216, http://www.moss-stock-center.org) 60, 61 was precipitated with TCA as described in 61. Sample preparation and subsequent SDS/PAGE as well as mass spectrometry were performed as described in 62. Raw data processing and database search were performed against the V1.6 protein models (http://www.cosmoss.org) using Mascot software (Matrix Science, Boston, MA, USA).

### *Physcomitrella patens* moss material and growth conditions

For all experiments, *P. patens* (Hedw.) B.S. ecotype ‘Gransden 2004′ was used. This is the same strain that was used for genome sequencing 63 and was deposited at the International Moss Stock Centre (IMSC, http://www.moss-stock-center.org) under accession number 40001. The Δ*PpBBE1* lines described in this study were cryopreserved according to 64 and are available from the International Moss Stock Center (IMSC) with the following accessions: 40829 for Δ*PpBBE1#80*, 40830 for Δ*PpBBE1#83*, and 40831 for Δ*PpBBE1#100*.

The material was axenically cultured in Knop medium (250 mg·L^−1^ KH_2_PO_4_, 250 mg·L^−1^ KCl, 250 mg·L^−1^ MgSO_4_ × 7 H2O, 1 g·L^−1^, Ca(NO_3_)_2_ × 4 H_2_O, and 12.5 mg·L^−1^ FeSO_4_ × 7H_2_O, pH 5.8; 65) supplemented with microelements 66 cultivated in a growth chamber under controlled conditions (25 °C) with a 16 h : 8 h, light:dark regime at a light intensity of 70 μmol·m^−2^·s^−1^ as described in 67. For supplementation of media with carbohydrates, cellobiose and glucose were added to a final concentration of 0.5% (w/v). For salt stress experiments, NaCl was added to the solid culture media in concentrations of 500 mm, 750 mm, and 1 m. For the adjustment of dry weight in *P. patens*, 10 mL of liquid culture was removed prior to subculturing and filtered through gauze (Miracloth, Calbiochem, Schwalbach, Germany). The dry weight was determined after drying the sample at 105 °C for 2 h. For the induction of sporophytes, the Petri dishes were transferred to inducing conditions according to 68. For dark treatment, protonema in liquid medium were grown for 3 weeks, whereas gametophores on solid medium were incubated in a light‐tight box in a growth chamber for 4–6 weeks. Additionally, spot inocula of protonema were grown in darkness for 4 weeks either directly after inoculation or after preculture under standard light conditions for 2 weeks.

### Generation and validation of Δ*PpBBE* lines

For construction of the *PpBBE1*‐knockout construct, genomic DNA was isolated according to 69 and used as template in an overlap extension PCR. Fragments of exon 1 and exon 3, together with the respective ~ 850 bp flanking regions, were amplified in separate PCR reactions, with the reverse primer for exon 1 (first 38 bp) and the forward primer for exon 3 (last 37 bp) carrying an overlapping sequence with the flanking regions of the ntpII selection cassette, which should replace the missing gene fragment (Fig. 9A, Table 4). In a second PCR reaction, all three fragments were fused, before subcloning the newly generated DNA to pJET and transforming the recombinant plasmid with *E. coli* top 10 cells for further amplification. Since sequencing of the subcloned DNA revealed a mutation, the error was removed in an additional mutagenesis PCR reaction (Primers see Table 4).

**Table 4:** Primers used for the generation of the Δ*PpBBE1* strain

Primer	Sequence
PpE1_fwd	5′‐ CGTGGCATGCATGCCTAAG‐3′
PpE1_rev	5′‐CGTGCTCCACCATGTTGACCGCCGCAGTAACCCTAAGCACTG‐3′
Pp_neo_fwd	5′‐CAGTGCTTAGGGTTACTGCGGCGGTCAACATGGTGGAGCACG ‐3′
Pp_neo_rev	5′‐CTTTGGGGGAAGTTGAAGACGTGCAGGTCACTGGATTTTGGTTTTAG ‐3′
PpE3_fwd	5′‐CTAAAACCAAAATCCAGTGACCTGCACGTCTTCAACTTCCCCCAAAG ‐3′
PpE3_rev	5′‐ CACCCTGTTGAAAATTAATTAATATTATTAAATCCTAG ‐3′
Pp_mut_fwd	5′‐CAGGCTCAAGGCGCGTATGCCCGACGGCG‐3′
Pp_mut_rev	5′‐CGCCGTCGGGCATACGCGCCTTGAGCCTG‐3′

Prior to the transformation procedure, the *PpBBE1*‐knockout construct was cut from the pJET vector using *Sph*I and *Pac*I. Protoplast isolation, transformation, and regeneration to obtain stable knockout plants were performed as previously described 23 using 40–50 μg of DNA. Selection was done in two successive cycles by transferring the cultures to solid media complemented with 25 mg·L^−1^ G418 (Promega, Mannheim, Germany)—release period of 2 weeks between the two selections as described in 23. Plants were screened for correct 5′‐ and 3′‐integration of the knockout construct via direct PCR 24 using the primer combinations BBE_5′Int_f and RT1 or RT4 and BBE_3′Int_r, respectively. As a positive control for successful extraction of DNA, the primers EF1α_fw and EF1α_rev were used. The loss of transcript in the putative Δ*PpBBE1* lines was verified by direct PCR, using the primer couple BBE_E2_f and BBE_E3I_r, which generates a transcript of 546 bp length in WT. As control for successful cDNA synthesis, C45_fwd und C45_rev were used, amplifying a part of the constitutively expressed mRNA of the ribosomal protein L21.

To estimate the number of integrations of *PpBBE1*‐targeting constructs, a qPCR‐based method 26 was used. The 5′‐HR region and the 3′‐HR region were amplified using the primer couples qPCR_BBE_5HR_fw and qPCR_BBE_5HR_rev and qPCR_BBE_3HR_fw and qPCR_BBE_3HR_rev, respectively, whereas transcripts of the ntpII selection cassette and the single copy gene pCLF (internal control) were generated using the primer couples q_npt_fw and q_npt_rev and pCLF_5915_qf and pCLF_5981_qr or pCLF_7739_qf and pCLF_7804_qr, respectively. From these results, the copy number of *Pp*BBE1‐knockout genes could be determined.

As control lines, a WT strain and a line with a confirmed single integration of the nptII selection cassette were used. The complete list of primers used for the cloning of the *PpBBE1*‐targeting construct and molecular validation of the transgenic plants is shown in Table 5.

**Table 5:** Primers used for the cloning of the *PpBBE1*‐targeting construct and the subsequent molecular validation of the transgenic plants

Purpose	Name	Sequence
5′‐integration direct PCR	BBE_5′Int_f	5′‐TCGACTACCAGCTTCTTGTGC‐3′
	RT1	5′‐TGTCGTGCTCCACCATGTTG‐3′
3′‐integration direct PCR	RT4	5′‐GTTGAGCATATAAGAAACCC‐3′
	BBE_3′Int_r	5′‐ACCTTGTCAAGGAGTGATG ‐3′
Control for DNA direct PCR	EF1α_fw	5′‐AGCGTGGTATCACAATTGAC‐3′
	EF1α_rev	5′‐GATCGCTCGATCATGTTATC‐3′
Loss of transcript RT‐PCR	BBE_E2_f	5′‐TGTCGCTCAAGTGCAGAATG‐3′
	BBE_E3I_r	5′‐TCTTGAACAACACCGCCTTG‐3′
cDNA control RT‐PCR	C45_fwd.	5′‐GGCTGGTCATGGGTTGCG‐3′
	C45_rev.	5′‐GAGGTCAACTGTCTCGCC‐3′
qPCR copy number nptII	q‐nptfw	5′‐GGCTATTCGGCTATGACTGG‐3′
	q‐nptrev	5′‐CAGGTCGGTCTTGACAAAAAG‐3′
qPCR copy number 5′HR	qPCR_BBE_5HR_fw	5′‐CAGTATTCTATTTGTTGCAGTTTCTCA‐3′
	qPCR_BBE_5HR_rev	5′‐GCTTCACGTTAGGCTGATTGA‐3′
qPCR copy number 3′HR	qPCR_BBE_3HR_fw	5′‐GGATGAACTGTAGAGAAATACTTCGAC‐3′
	qPCR_BBE_3HR_rev	5′‐AGTAGTGACCGTTTCTTAATGAAGTTT‐3′
qPCR copy number control	PpCLF_5915_qf	5′‐AGCAATGTCCGTGCCTACTT‐3′
	PpCLF_5981_qr	5′‐TTGTAAGAATCACTCACCCACAG‐3′
qPCR copy number control	PpCLF_7739_qf	5′‐GTATTGGCGATCCCACTCTT‐3′
	PpCLF_7804_qr	5′‐GCATAAAATAGGTCACAGATTGAGG‐3′

As the DNA content of *Physcomitrella* is known, haploidy of the Δ*PpBBE1* lines could be confirmed in a fluorescence‐based assay. Nuclei prepared from 10 to 30 mg freshly chopped protonema material were stained with a 4′,6‐diamidino‐2‐phenylindole (DAPI)‐containing buffer 25 and analyzed using a Cyflow® Space flow cytometry system (Partec, Münster, Germany) as described in 70.

### Qualitative and quantitative determination of cellobiose and glucose

In order to qualitatively assay the cellobiose and glucose content of solid media, plates were stained with a solution of 0.1% (w/v) congo red. After washing with a 0.5 m NaCl solution for five times, for better visualization of lighter stained zones, the pH was adjusted to 2. Benedict's test, using 1 mL of the sample and 200 μL of Benedict's reagent, performed according to 71, was used to qualitatively analyze the liquid culture supernatants. Here, a color change from blue to red, caused by the precipitation of copper sulfate, indicates the presence of reducing sugars in the solution. For quantification of cellobiose and glucose in the liquid media, a DNS assay was performed as described 72—250 μL of sample was mixed with 250 μL of 0.005 m sodium acetate buffer pH 4.8 and 750 μL of DNS reagent. Photometric quantification was done at 575 nm for all samples and appropriate standard dilution series for each sugar. Additionally, sugars were quantified using the glucose test strips of the Reflectoquant system (Merck).

### Image acquisition

Images were acquired with an ICc1 or Mrc5 CCD camera (Zeiss, Oberkochen, Germany) at an Olympus SZX7 binocular microscope (Olympus, Tokyo, Japan) or an Axiovert S100 (Zeiss). axiovision Software Version 4.8 (Zeiss) was used for imaging.

## Author contributions

PM initiated the project; MT, RR, GW, BD, and PM designed the experiments and interpreted the data. MT expressed and purified *Pp*BBE1 and performed analytical, biochemical, and kinetic experiments. MT and AW crystallized proteins and determined the 3D structure of *Pp*BBE1. GW analyzed organization and expression of the *Pp*BBEs and generated knockout moss mutants. GW and JK analyzed the moss mutants. SNWH analyzed the secretion of *Pp*BBE1 into the moss culture medium. JN performed docking; MT, AW, and PM wrote the manuscript with the help of RR and GW.

## Conflict of interest

The authors declare no conflict of interest.

## Supporting information


**Fig. S1**. NMR analyses of the product formed in the enzymatic reaction of *Pp*BBE1 with cellobiose.
