# Supplementary material for: The single berberine bridge enzyme homolog of Physcomitrella patens is a cellobiose oxidase
Source: FEBS J. 2018 Apr 19;285(10):1923–43. doi: 10.1111/febs.14458 (PMC6001459; doi:10.1111/febs.14458)
Supplement: Supplementary file 1 — Fig. S1. NMR analyses of the product formed in the enzymatic reaction of PpBBE1 with cellobiose. [file FEBS-285-1923-s001.zip › febs14458-sup-0001-FigS1.pdf]

FEBS PRESS
science publishing by scientists
WILEY
The FEBS Journal
www.febsjournal.org

The single berberine bridge enzyme homolog of *Physcomitrella patens* is a cellobiose oxidase

Marina Toplak, Gertrud Wiedemann, Jelena Ulicevic, Bastian Daniel, Sebastian N. W. Hoernstein, Jennifer Kothe, Johannes Niederhauser, Ralf Reski, Andreas Winkler and Peter Macheroux

DOI: 10.1111/febs.14458

SUPPORTING INFORMATION

The single berberine bridge enzyme homolog of *Physcomitrella patens* is a cellobiose oxidase

Marina Toplak,[1] Gertrud Wiedemann,[2] Jelena Ulićević,[1] Bastian Daniel,[1] Sebastian N. W. Hoernstein,[2] Jennifer Kothe,[2] Johannes Niederhauser,[1] Ralf Reski,[2,3] Andreas Winkler[1] and Peter Macheroux[1]

[1]Institute of Biochemistry, Graz University of Technology, Petersgasse 12/2, A-8010 Graz, Austria

[2] Plant Biotechnology, Faculty of Biology, University of Freiburg, Schaenzlestrasse 1, D-79104 Freiburg, Germany

[3]BIOSS Centre for Biological Signalling Studies, University of Freiburg, Schaenzlestrasse 18, D-79104 Freiburg, Germany

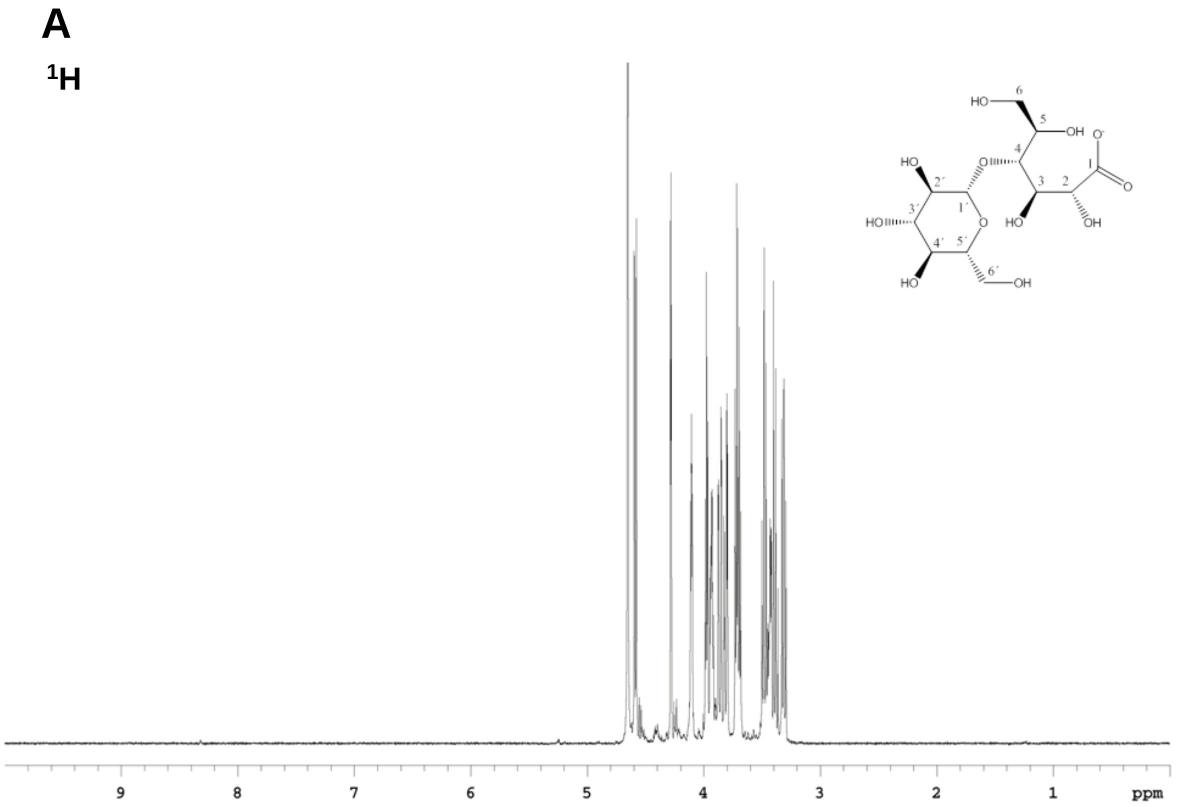
A
1H
9
8
7
6
5
4
3
2
1
ppm

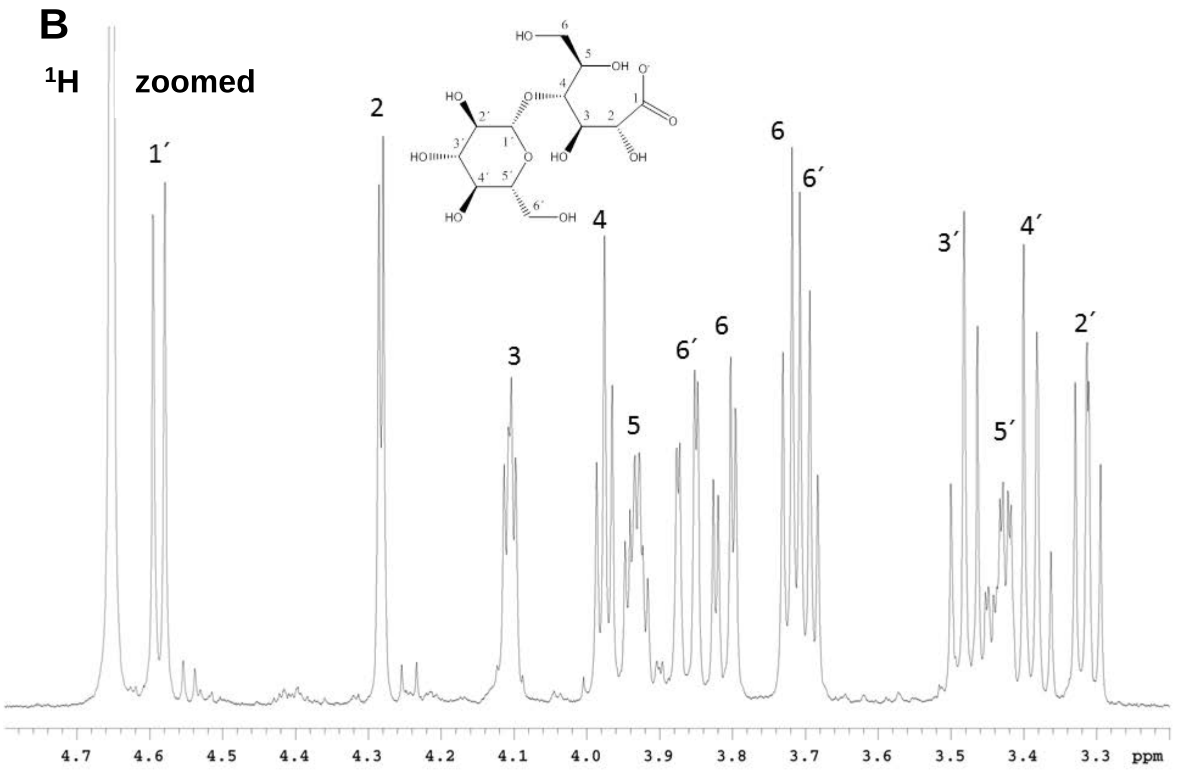
B
1H zoomed
1´
2
3
4
5
6´
6
6
6´
3´
4´
5´
2´
4.7
4.6
4.5
4.4
4.3
4.2
4.1
4.0
3.9
3.8
3.7
3.6
3.5
3.4
3.3
ppm

C

^{13}C

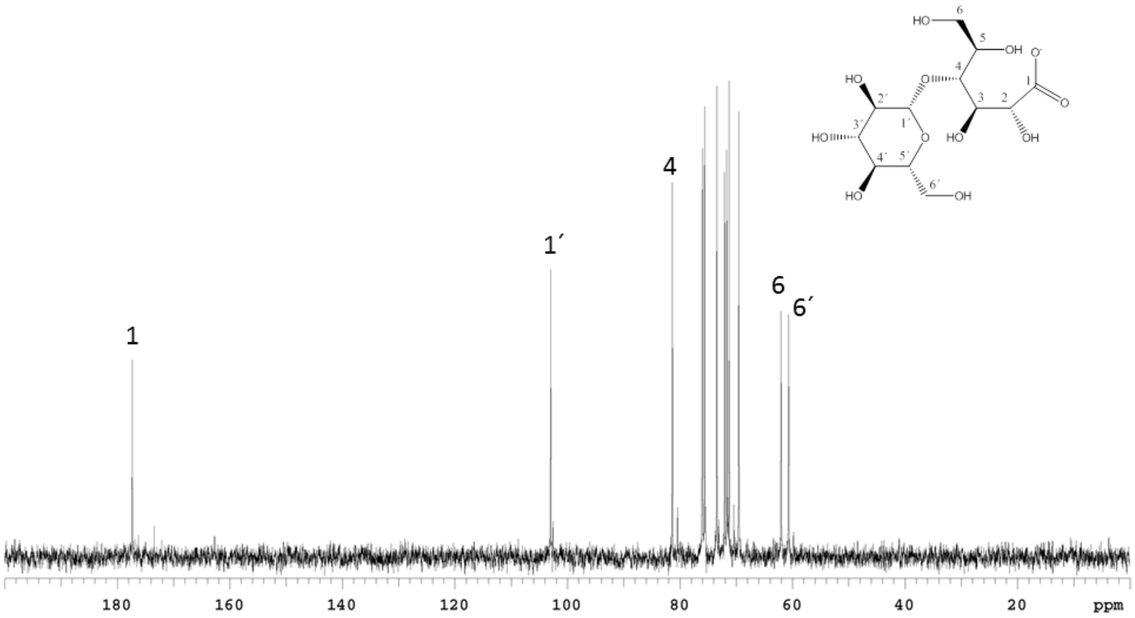

D

^{13}C zoomed

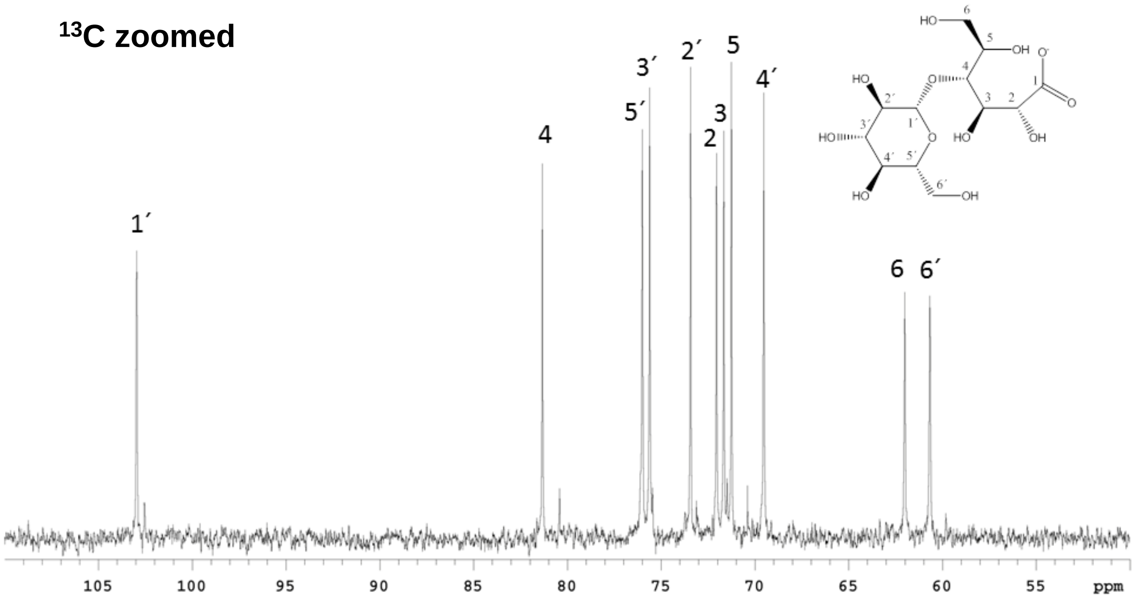

E

COSY

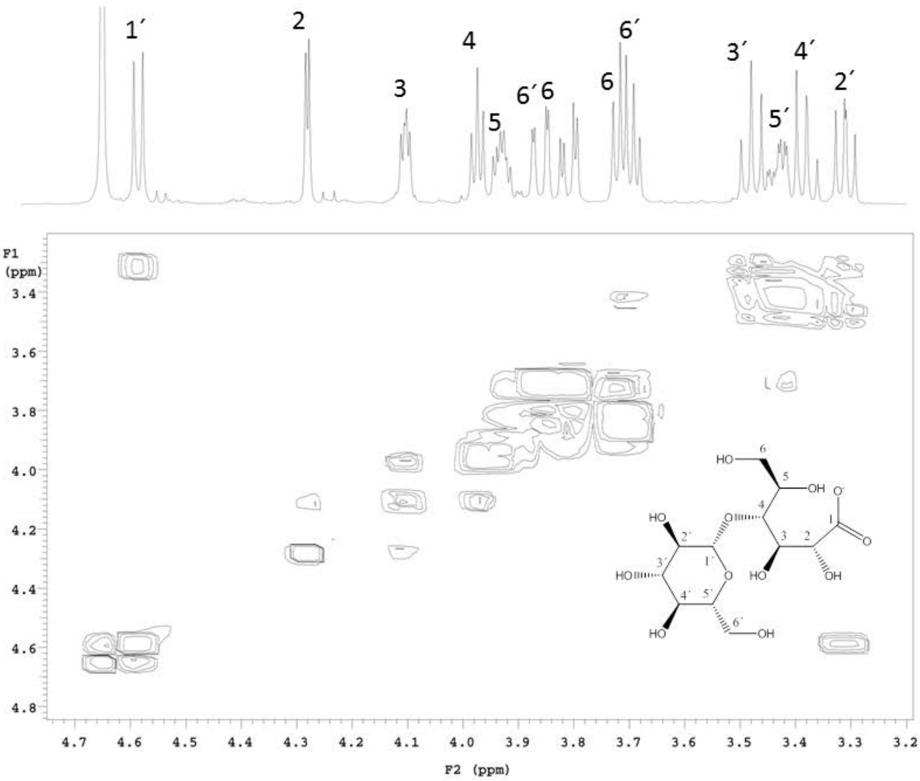
1´
2
3
4
5
6´6
6
6´
3´
4´
5´
2´
F1 (ppm)
3.4
3.6
3.8
4.0
4.2
4.4
4.6
4.8
4.7 4.6 4.5 4.4 4.3 4.2 4.1 4.0 3.9 3.8 3.7 3.6 3.5 3.4 3.3 3.2
F2 (ppm)

F

HSQC

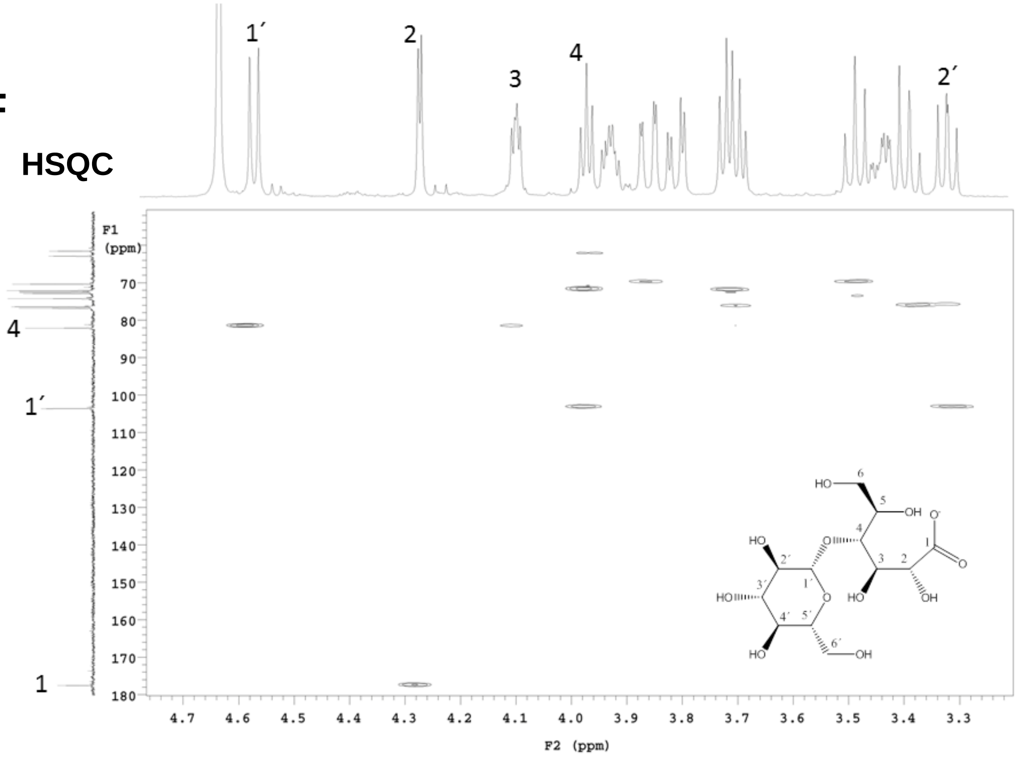
1´
2
3
4
2´
F1 (ppm)
70
80
90
100
110
120
130
140
150
160
170
180
4
1´
1
4.7 4.6 4.5 4.4 4.3 4.2 4.1 4.0 3.9 3.8 3.7 3.6 3.5 3.4 3.3
F2 (ppm)

G

HMBC

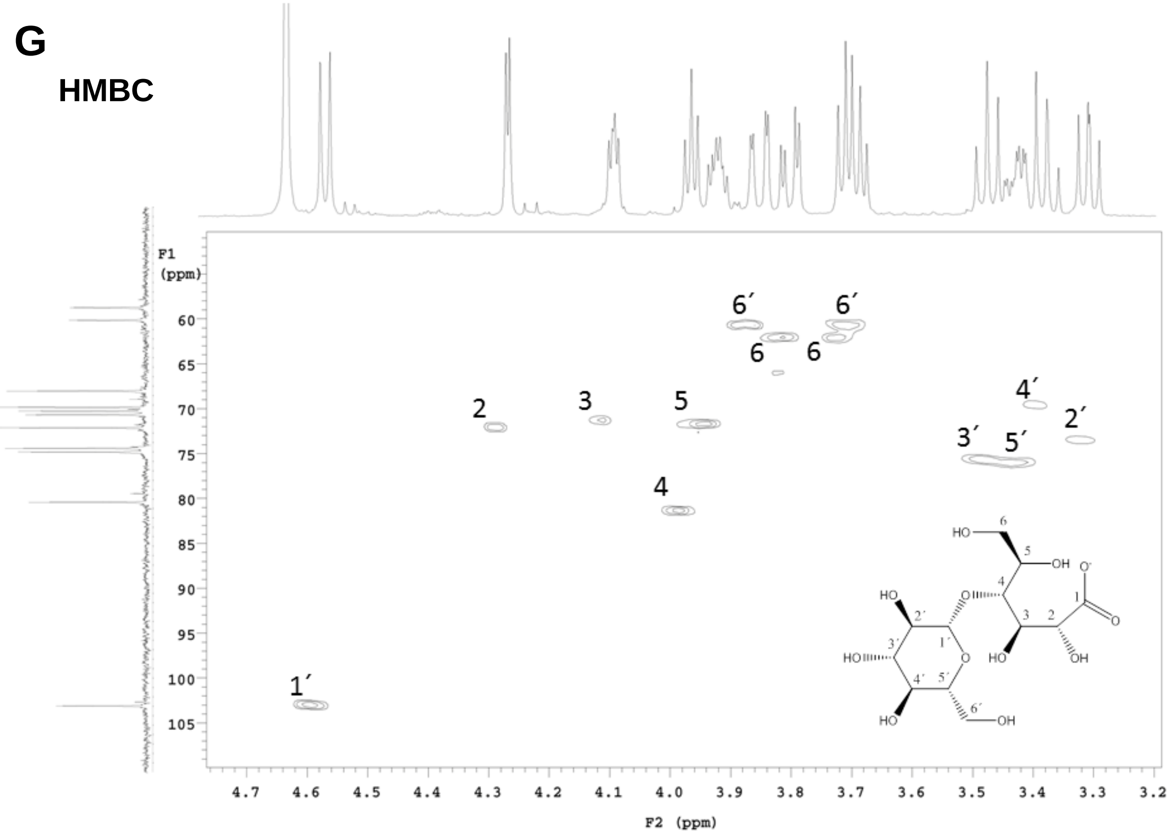

Supplementary Figure S1: NMR-analyses of the product formed in the enzymatic reaction of *Pp*BBE1 with cellobiose: ^{1}H- (panel A: full spectrum, panel B: zoomed), ^{13}C- (panel C: full spectrum; panel D: zoomed), COSY (panel E), HSQC (panel F) and HMBC (panel G) spectra were recorded in D_2O to clearly identify the product of the enzymatic turnover of cellobiose by *Pp*BBE1.
